# The HMGB1 protein induces a metabolic type of tumour cell death by blocking aerobic respiration

**DOI:** 10.1038/ncomms10764

**Published:** 2016-03-07

**Authors:** Georg Gdynia, Sven W. Sauer, Jürgen Kopitz, Dominik Fuchs, Katarina Duglova, Thorsten Ruppert, Matthias Miller, Jens Pahl, Adelheid Cerwenka, Markus Enders, Heimo Mairbäurl, Marcin M. Kamiński, Roland Penzel, Christine Zhang, Jonathan C. Fuller, Rebecca C. Wade, Axel Benner, Jenny Chang-Claude, Hermann Brenner, Michael Hoffmeister, Hanswalter Zentgraf, Peter Schirmacher, Wilfried Roth

**Affiliations:** 1 Institute of Pathology, Department of Surgical Pathology, University of Heidelberg, 69120 Heidelberg, Germany; 2 German Cancer Research Center, Clinical Cooperation Unit Molecular Tumor Pathology, 69120 Heidelberg, Germany; 3 Division of Inborn Metabolic Diseases, Department of General Pediatrics, University Children's Hospital, 69120 Heidelberg, Germany; 4 German Cancer Research Center, Boveri Junior Research Group Innate Immunity, 69120 Heidelberg, Germany; 5 Institute of Inorganic Chemistry, Research Group Enders, University of Heidelberg, 69120 Heidelberg, Germany; 6 Medical Clinic VII, Department of Sports Medicine, University of Heidelberg, and Translational Lung Research Center (TLRC), member of the German Center for Lung Research (DZL), 69120 Heidelberg, Germany; 7 German Cancer Research Center, Division of Immunogenetics, Tumour Immunology Program, 69120 Heidelberg, Germany; 8 Molecular and Cellular Modeling Group, Heidelberg Institute for Theoretical Studies (HITS), Department of Molecular and Cellular Modeling (MCM), 69118 Heidelberg, Germany; 9 Center for Molecular Biology (ZMBH), Molecular and Cellular Modeling (MCM), DKFZ-ZMBH Alliance, Heidelberg University, Heidelberg 69120, Germany; 10 Interdisciplinary Center for Scientific Computing (IWR), Heidelberg University, 69120 Heidelberg, Germany; 11 German Cancer Research Center, Division of Biostatistics, 69120 Heidelberg, Germany; 12 Unit of Genetic Epidemiology, German Cancer Research Center, Division of Cancer Epidemiology, 69120 Heidelberg, Germany; 13 University Cancer Center Hamburg (UCCH), University Medical Center Hamburg- Eppendorf, 20246 Hamburg, Germany; 14 German Cancer Research Center (DKFZ), Division of Clinical Epidemiology and Aging Research, 69120 Heidelberg, Germany; 15 German Cancer Research Center (DKFZ) and National Center for Tumor Diseases (NCT), Division of Preventive Oncology, 69120 Heidelberg, Germany; 16 German Cancer Research Center, Division of Monoclonal Antibodies, 69120 Heidelberg, Germany; 17 Institute of Pathology, Department of Surgical Pathology, University Medical Center Mainz, University of Mainz, 55131 Mainz, Germany

## Abstract

The high-mobility group box 1 (HMGB1) protein has a central role in immunological antitumour defense. Here we show that natural killer cell-derived HMGB1 directly eliminates cancer cells by triggering metabolic cell death. HMGB1 allosterically inhibits the tetrameric pyruvate kinase isoform M2, thus blocking glucose-driven aerobic respiration. This results in a rapid metabolic shift forcing cells to rely solely on glycolysis for the maintenance of energy production. Cancer cells can acquire resistance to HMGB1 by increasing glycolysis using the dimeric form of PKM2, and employing glutaminolysis. Consistently, we observe an increase in the expression of a key enzyme of glutaminolysis, malic enzyme 1, in advanced colon cancer. Moreover, pharmaceutical inhibition of glutaminolysis sensitizes tumour cells to HMGB1 providing a basis for a therapeutic strategy for treating cancer.

## Abstract (web summary)

HMBG1 is a protein expressed in natural killer cells and is important in immunosurveillance. In this study, the authors show that HMGB1 binds to and inhibits PKM2, resulting in a block in aerobic glycolysis and ultimately cell death.

## Graphical abstract

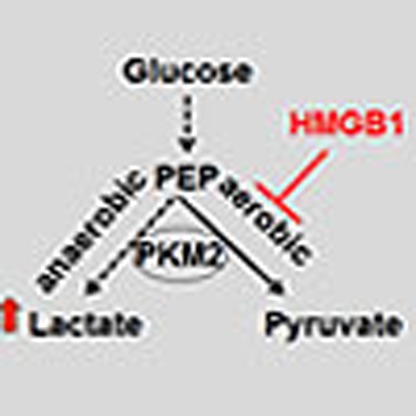

The high-mobility group box 1 (HMGB1) protein is a ubiquitously expressed cytokine known for its pro-inflammatory effects on release from macrophages^1^,^2^. In the setting of cancer, HMGB1 signalling through its innate immune system receptors TLR2 and TLR4 (toll-like receptors 2 and 4) is important for an antitumour immune response in breast cancer patients. A TLR4 single-nucleotide polymorphism reduces the interaction between HMGB1 and TLR4 thereby inhibiting antigen presentation which is associated with a poor prognosis of breast cancer patients^3^. Furthermore, the release of high amounts of HMGB1, in particular from natural killer (NK) cells, is pivotal for dendritic cell activation^4^ and chemotaxis^5^. In addition, HMGB1 exhibits striking antimicrobial activity resulting in rapid killing of bacteria^6^.

However, endogenous HMGB1 is also intricately involved in the energy metabolism of cells and organs. HMGB1 knock-out mice are unable to utilize glycogen storage pools in hepatocytes and die due to perinatal hypoglycaemia. Glucose temporarily rescues the animals, but the mice succumb several days later due to severe atrophy of inner organs, muscle and fatty tissue^7^. *Ex vivo* incubation of murine muscle tissue with HMGB1 leads to rapid exhaustion of muscle fibres, and elevated HMGB1 concentrations are found in the myoplasm of patients suffering from polymyositis^8^. In summary, both lack and excess of HMGB1 severely affects cellular energy metabolism.

Recently, we described that HMGB1 induces a distinct form of necrotic cell death in cancer cells which differed from the classical cell death entities known so far^9^. One of the main targets of HMGB1 turned out to be the mitochondrial energy metabolism as tumour cells devoid of a functioning mitochondrial respiratory chain were resistant to HMGB1 cytotoxicity. In this study, we investigated whether the cytotoxic activity of HMGB1 plays a role in antitumour defense mechanisms. Our data provide evidence that the innate immune system employs specific forms of ‘metabolic weapons' to target cancer cells. HMGB1 physically interacts with the pyruvate kinase (PK) isoform M2 resulting in a rapid blockage of glucose-dependent aerobic respiration. Thus, secreted HMGB1 can kill cancer cells by causing a brisk metabolic shift restricting their energy supply to glycolysis. This establishes a link between innate tumour defense and tumour metabolism.

## Results

### NK cell HMGB1 induces cell death in colorectal cancer

**Figure 1:**
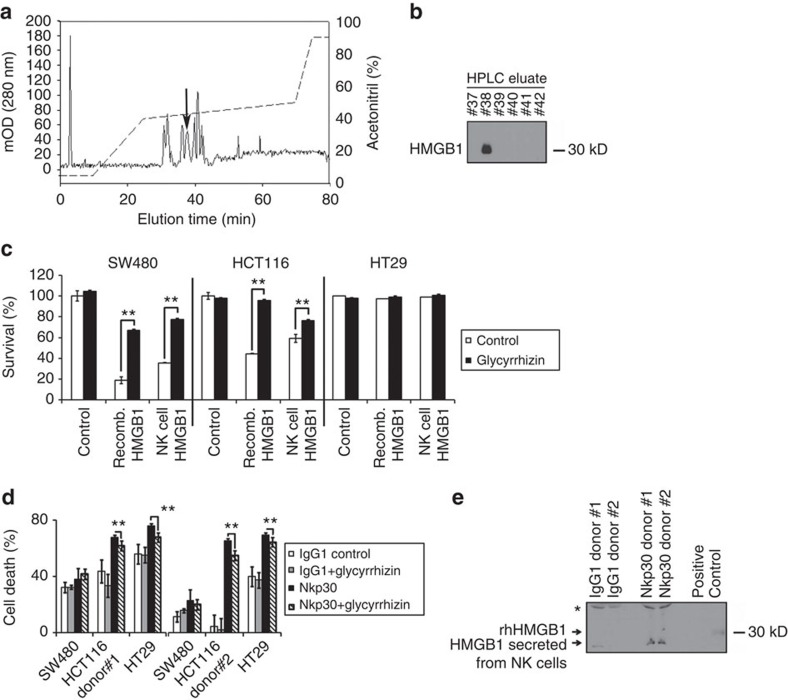
HMGB1 is released from NK cells and induces cell death in CRC. (**a**) HMGB1 was purified from NK-92 Cl cells by chromatography (*n*=2). Arrow=HMGB1 containing fraction (eluate #38). (**b**) Immunoblot showing the membrane containing eluates 37–42. A specific HMGB1 band at 30 kD was detected only in eluate #38 (Supplementary Fig. 1C). (**c**) Cytotoxicity assay after 72 h incubation with HMGB1 (*n*=3). A 1:40 dilution of the purified HMGB1 (#38) was used (corresponding to ∼16 nM). Recombinant human HMGB1 was used at 80 nM. (**d**) Supernatants from activated human peripheral blood NK cells (cross-linked with anti-Nkp30 antibody) from two donors were tested for their cytotoxic capacity in a crystal violet assay (72 h, *n*=3). Glycyrrhizin (200 μM) was used as an inhibitor of HMGB1. (**e**) Immunoblot of the supernatants used in **d**. HMGB1 was specifically secreted on Nkp30 crosslinking. *, non-specific band. **P<0.002 (*t*-test). Error bars represent s.d.

Given the cytotoxic activity of recombinant human HMGB1 protein on cancer cells^9^, we sought to examine the cellular effects of immune cell-derived endogenous HMGB1. To this end, we isolated HMGB1 from the cytosolic granules of the NK cell line NK-92 Cl by high-performance liquid chromatography (HPLC; Fig. 1a, Supplementary Figs 1A,B). Elution of HMGB1 was confirmed by immunoblot analysis (Fig. 1b). Both NK cell-derived HMGB1 and, as a comparison, recombinant human HMGB1 efficiently killed SW480 and HCT116 colorectal cancer (CRC) cells (Fig. 1c), respectively. The observed cell death was specific for HMGB1 since glycyrrhizin, an inhibitor of HMGB1, significantly blocked its cytotoxic effects. In contrast, HT29 cells were resistant to low to intermediate HMGB1 concentrations (16–80 nM). Higher concentrations (80 or 160 nM) of NK cell-derived HMGB1 exerted higher cytotoxicity than recombinant HMGB1 as assessed in side-by-side cytotoxicity experiments (Supplementary Fig. 1D).

**Figure 2:**
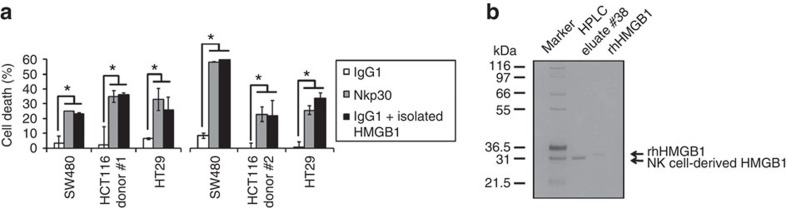
HMGB1 from human blood donors induces cell death in CRC. (**a**) HPLC-purified HMGB1 (80 nM, 24 h, *n*=3) from the supernatant of Nkp30-stimulated blood donor NK cells was diluted in the IgG1 control supernatant and shows substantial cytotoxicity. (**b**) Silver gel showing purity of HMGB1 in eluate #38 (0.5 μg protein loaded). *P<0.05 (*t*-test). Error bars represent the s.d.

On stimulation of activated human peripheral blood NK cells from healthy blood donors by agonistic anti-NK cell p30-related protein (Nkp30) mAbs, the NK cell-dependent cytotoxic effect on HT29 and HCT116 colon cancer cells was diminished by the HMGB1-specific inhibitor glycyrrhizin, indicating that HMGB1 was partly mediating the NK cell-triggered tumour cell death (Fig. 1d). Secretion of HMGB1 from NK cells was confirmed by immunoblot (Fig. 1e). In an alternative experimental set-up with independent blood donors we (1) diluted NK cell supernatants to decrease non-specific cytotoxicity (in the immunoglobulin G 1 (IgG1) control group) and (2) isolated HMGB1 by HPLC from the Nkp30-stimulated supernatants and added it to the IgG1 control supernatants resulting in substantial cytotoxicity, thereby confirming the specific capability of NK-derived HMGB1 to kill cancer cells (Fig. 2a). A silver staining gel of eluate #38 confirmed that HMGB1 was isolated with high purity (single band at ∼30 kD, Fig. 2b).

Moreover, high levels of Interferon-γ were detected in the supernatant, indicating activation of NK cells by the agonistic anti-NKp30 mAb (Supplementary Fig. 1E). As a control experiment, treatment with HMGB1 did not generally alter the transcriptional or translational regulation in cells (Supplementary Figs 1F,G). Taken together, NK cell-derived HMGB1 protein induces cell death in CRC cells.

### HMGB1 inhibits aerobic respiration *in vitro* and *ex vivo*

**Figure 3:**
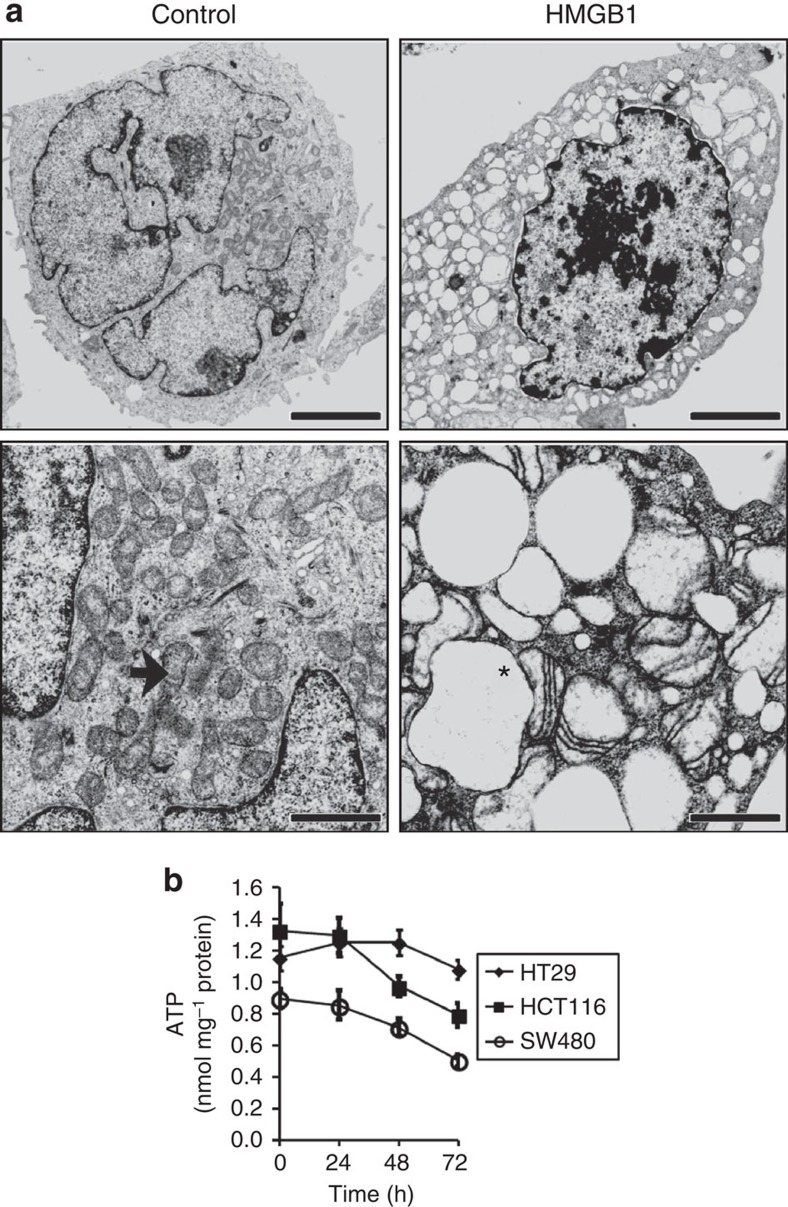
HMGB1 treatment results in formation of giant mitochondria and ATP loss. (**a**) Electron microscopy showing the ultrastructure of mitochondria in CRC cells (SW480) treated with HMGB1 (24 h, 160 nM). Upper bars, 5 μm; lower bars, 1 μm; black arrow, normal mitochondria; asterisk, giant mitochondria. (**b**) ATP Luciferase-assay after incubation with 80 nM HMGB1 for the indicated times (*n*=6 for SW480 and HCT116, *n*=3 for HT29). Error bars represent s.d.

**Figure 4:**
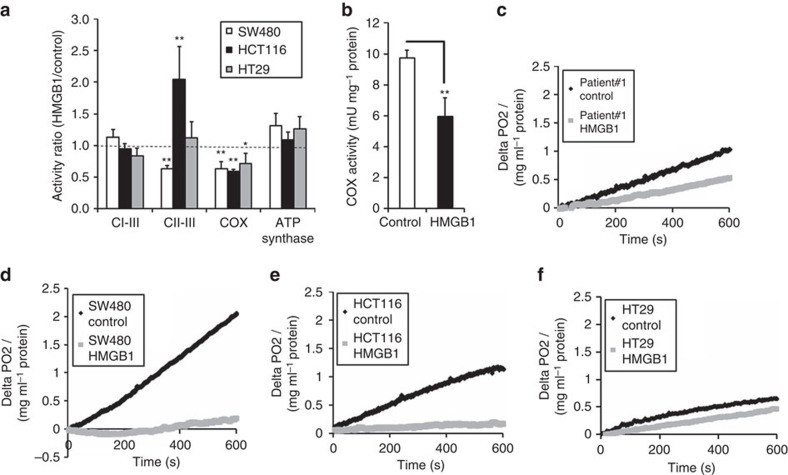
HMGB1 inhibits mitochondrial respiration. (**a**) Activities of respiratory chain complexes measured in mitochondrial fractions of CRC cell lines after treatment with HMGB1 (80 nM, 24 h, *n*=5). (**b**) Tissue slices were generated from a fresh surgical human colon carcinoma specimen and treated with HMGB1 (160 nM, 72 h). After homogenization of tissue slices, COX activity was measured in the mitochondrial fractions (*n*=8). (**c**–**f**) CRC cells and tissue slices were treated with HMGB1 as described in **a** and **b**, respectively. Then, cyanide sensitive respiration was measured. *P<0.05, **P<0.001 (*t*-test). Error bars represent s.d.

The HMGB1-mediated cell death was characterized by formation of giant mitochondria (Fig. 3a) and a substantial decrease of ATP in HMGB1-sensitive (SW480) and HMGB1 partly resistant (HCT116) cancer cells, but not in HMGB1-resistant HT29 cells (Fig. 3b). Due to the observed loss of energy equivalents and the altered mitochondrial morphology, we examined whether HMGB1 affects the main ATP generating pathways, oxidative phosphorylation (OXPHOS) and glycolysis. HMGB1 treatment resulted in significant lower activity levels of cytochrome *c* oxidase (COX) which is vital for oxygen-derived ATP generation (Fig. 4a). Electron flow from complex I–III was unchanged, whereas coupled complex II and III activity was decreased in the HMGB1-sensitive cells (SW480) and maintained or even upregulated in the partly HMGB1-resistant cell line HCT116 and the HMGB1-resistant cell line HT29. ATP synthase activity was not diminished supporting the hypothesis that the decrease of intracellular ATP was caused by inhibition of energy metabolism upstream of the respiratory chain. Next, we confirmed our *in vitro* monolayer cell-culture-based results in an alternative model accounting for the *in vivo* complexity of human CRC tissue using 300-μm-thick slices from fresh tumour tissue of CRC patients. HMGB1 treatment decreased the turn-over of oxygen as demonstrated by a potent inhibition of COX activity in the primary tumour tissue (Fig. 4b). Consistently, HMGB1 strongly decreased mitochondrial oxygen consumption in CRC tissue (Fig. 4c). A similar effect was observed in cultured colon cancer cells, where the inhibition of mitochondrial oxygen consumption was pronounced in HMGB1-sensitive SW480 cells and in partly HMGB1-resistant HCT116 cells (Fig. 4d,e), whereas mitochondrial respiration of HMGB1-resistant HT29 cells was only slightly reduced by HMGB1 (Fig. 4f). These results indicate that HMGB1 inhibits aerobic respiration in colorectal carcinoma cells.

### HMGB1 controls glycolysis by inhibition of PKM2

**Figure 5:**
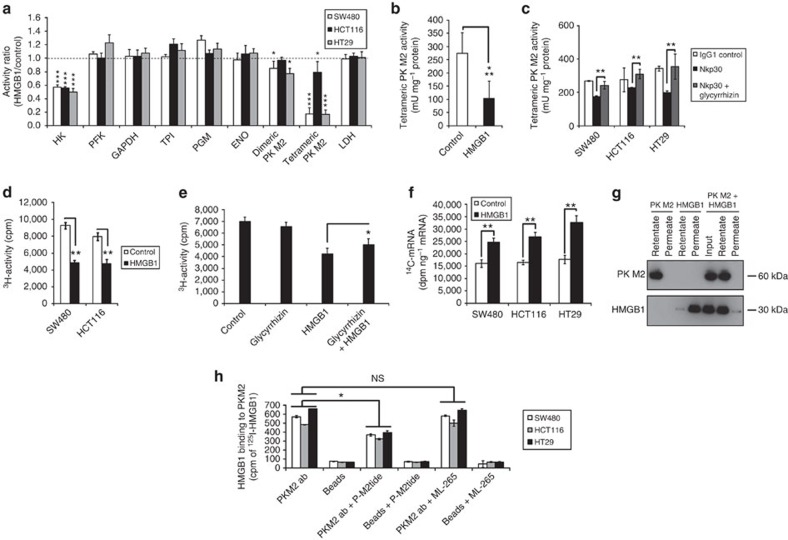
HMGB1 blocks glycolysis by interfering with PK M2. (**a**) Activities of glycolytic enzymes measured in cytosolic fractions after treatment with HMGB1 (80 nM, 24 h, *n*=5). ENO, enolase; GAPDH, glyceraldehyde 3-phosphate dehydrogenase; HK, hexokinase; LDH, lactate dehydrogenase; PGM, phosphoglycerate mutase; PFK, phosphofructokinase; TPI, triose-phosphate isomerase. (**b**) Colon cancer tissue slices from a fresh surgical specimen were treated with HMGB1 (160 nM, 72 h). Tetrameric PK M2 activity was measured in eight homogenates. (**c**) PK M2 activity in CRC cells was measured after 24 h incubation with the supernatant derived from stimulated NK cells from blood donor #2 (Fig. 1d). Glycyrrhizin (200 μM) was used as a HMGB1 inhibitor (*n*=3). (**d**) 5-^3^H-glucose turn-over was assessed after treatment with HMGB1 (80 nM, 24 h, *n*=3). (**e**) The experiment using SW480 cells was performed as outlined in **d**. Glycyrrhizin (200 μM) was used as an inhibitor of HMGB1 (*n*=3). (**f**) Enrichment of ^14^C in the messenger RNA of crude extracts after treatment with HMGB1 (80 nM, 24 h, *n*=3). (**g**) Isolation of the PK M2-HMGB1 complex: ultrafiltration of a solution containing 2 μM human PK M2 and 2 μM human HMGB1. The filtrated PK M2-HMGB1 complex was exposed to western blotting. (**h**) Cells were treated with ^125^I-HMGB1 (80 nM, 24 h, *n*=3) with or without the PKM2 tetramer inhibitor P-M2tide (10 μM, 24 h) or PKM2 activator ML-265 (100 μM, 24 h). *P<0.05, **P<0.002, ***P<0.00008 (*t*-test). Error bars represent s.d.

Since aerobic respiration can be glucose-driven we studied the effect of HMGB1 on the activity of the major glycolytic enzymes. We observed a reduced activity of an isoform (M2) of PK after HMGB1 treatment (Fig. 5a) that is known to drive glucose-mediated respiration. HMGB1 specifically inhibited the tetrameric PK isoform PK M2 in all CRC cell lines tested as well as in *ex vivo* tissue slice cultures (Fig. 5a,b). Further experiments showed that HMGB1 containing supernatants from Nkp30-stimulated NK cells from a blood donor (Fig. 1 donor #2) also significantly inhibited the tetrameric PK M2 (Fig. 5c). Importantly, this inhibition was caused by HMGB1 since glycyrrhizin completely restored tetrameric PK M2 activity. Dimeric PK M2 activity was unchanged (data not shown). Glucose flux in HMGB1-treated cells was reduced at the enolase reaction step (Fig. 5d). The observed metabolic shift was partly reversed by co-treatment with the HMGB1 inhibitor glycyrrhizin (Fig. 5e). Moreover, HMGB1 treatment resulted in an increased flux of glycolytic intermediates into the pentose phosphate shunt (Fig. 5f). Consistent with the accumulation of glucose intermediates upstream of PK there was a strong increase in the hexokinase product glucose-6-phosphate that could explain the observed decrease in hexokinase activity by product inhibition (Fig. 5a, Supplementary Fig. 2). Supporting the results from the enzymatic tests, HMGB1 physically interacted with PK M2 (Fig. 5g) *in vitro*. Using ^125^I-labelled HMGB1 we could show specific binding of HMGB1 to PKM2 *in vivo* by immunoprecipitating PKM2 (Fig. 5h). Non-cytotoxic P-M2tide concentrations substantially inhibited binding of HMGB1 to PKM2 supporting our *in silico* results. These data implicate that HMGB1 binding competes with the P-M2tide PKM2 binding site, involving the K433 near the fructose 1,6-bisphosphate (FBP) pocket of PKM2. Importantly, the small molecule ML-265, an activator of PKM2, previously identified to bind to the dimer–dimer-interface of PKM2 (ref. 10) far away from the FBP-binding pocket) did not compete with HMGB1 binding to PKM2 (Fig. 5h).

### Allosteric inhibition of tetrameric PK M2 by HMGB1

**Figure 6:**
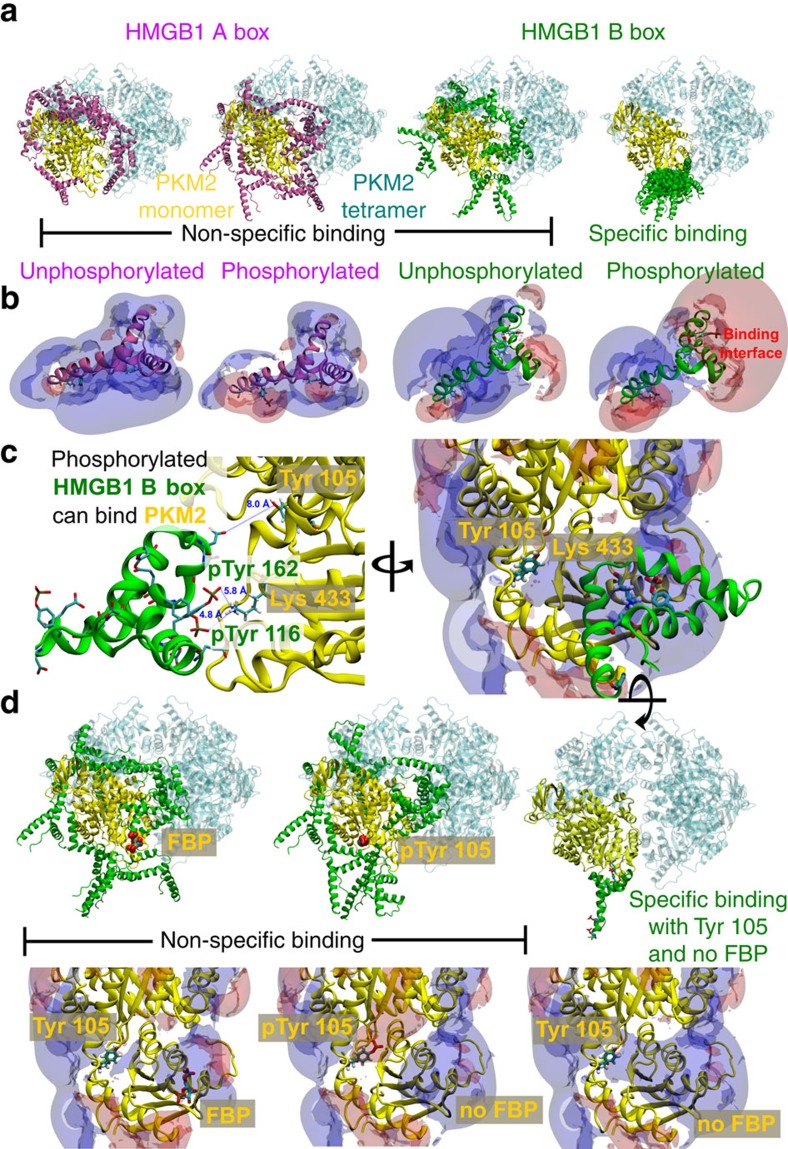
HMGB1 is an allosteric inhibitor of tetrameric PK M2. (**a**) The calculations were performed for the following HMGB1 constructs: leftmost: A box (magenta), tyrosines unphosphorylated; centre left: A box, tyrosines phosphorylated; centre right: B box (green), tyrosines unphosphorylated; rightmost: B box, tyrosines phosphorylated. PK M2 monomer, yellow; three additional PK M2 domains in the PK M2 tetramer, cyan. (**b**) Electrostatic isopotential contours (+1 kT/e: blue; −1 kT/e: red) of the HMGB1 A and B boxes with: leftmost) A box, unphosphorylated tyrosines; centre left) A box, phosphorylated tyrosines; centre right) B box, unphosphorylated tyrosines; rightmost) B box, phosphorylated tyrosines. (**c**) Left: distances from HMGB1 Box B residues pTyr 116 and pTyr 162 (numbering according to PDB file: 2YRQ, corresponding to residues 109 and 155, respectively, in the human sequence) to PK M2 K433 for the best ranked docked pose and of PK M2 Y105 to the nearest charged residue from the HMGB1 box B. Right: a rotated view with electrostatic isopotential contours (+1 kT/e: blue; −1 kT/e: red) of PK M2. (**d**) The calculations was performed as outlined in **a**, here in the presence of FBP (left) or with Tyr 105 phosphorylated (centre) or in the absence of FBP and with unphosphorylated Tyr 105 (right).

To characterize the inhibition of tetrameric PK M2 by HMGB1 in more detail, we performed *in silico* protein docking studies. The polyphosphorylated HMGB1 B box produced a single cluster of poses indicating specific binding to PK M2 (Fig. 6a). Specific binding was not observed when the same calculation procedure was applied to the unphosphorylated HMGB1 B box, or the polyphosphorylated or unphosphorylated HMGB1 A box (Fig. 6a). This was further supported by energetic analysis of the bound clusters which showed a strongly electrostatically driven binding for the polyphosphorylated HMGB1 B box, which is in contrast to a mainly hydrophobically driven binding typical of non-specific binding in the three other test cases (Fig. 6b). For the phosphorylated B Box, where specific binding is observed, a large region of negative electrostatic potential (red isopotential) was present in the vicinity of the binding interface, whereas the non-specific binding cases lacked such a region (Fig. 6b). Furthermore, the interaction involves K433 of PK M2 (Fig. 6c), previously shown to be involved in phosphotyrosine (pTyr) peptide binding near the FBP-binding pocket^11^, and in the regulation of PK M2 activity through controlling tetramerization^12^. There is variation of the electrostatic potential in the region surrounding the proposed HMGB1 binding site on binding of FBP or phosphorylation of Y105 (Fig. 6d). The decrease in the size of the positive electrostatic potential on binding FBP, or the introduction of negative electrostatic potential on phosphorylation of Y105 is likely to hinder binding of the negatively charged phosphate groups from the phosphorylated HMGB1 box B (Fig. 6d). These results support the hypothesis that HMGB1 is an allosteric inhibitor of the PK M2 tetramer.

Moreover, we could phenocopy the observed cell death using a known inhibitor of the PKM2 tetramer, a phosphotyrosine peptide called P-M2tide (Supplementary Fig. 3A). P-M2tide has previously been shown to bind near the FBP-binding pocket involving the interaction of K433 of PKM2 (ref. 11). Whereas P-M2tide induced substantial cell death, an activator of PKM2, the small molecule ML-265 was not able to induce cell death (Supplementary Fig. 3A). Penetration of the cell membrane was confirmed using ^125^I-labelled HMGB1 (ref. 9), showing a rapid (24 h) increase of cytosolic radioactivity (Supplementary Fig. 3B). Gain- and loss-of-function experiments for PKM2 using PKM2 siRNA/plasmid showed that downregulation of PKM2 sensitized the cells to HMGB1, whereas overexpression of PKM2 rendered them more resistant to HMGB1 (Supplementary Fig. 3C–E).

### Glycolysis and glutaminolysis mediate HMGB1 resistance

**Figure 7:**
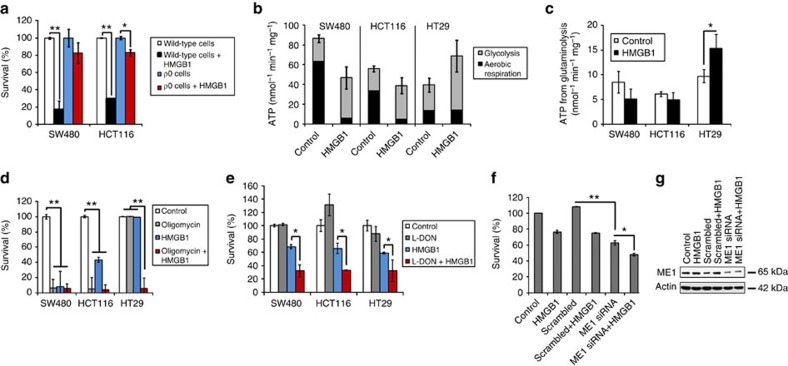
Glucose fermentation and glutaminolysis circumvent the HMGB1-triggered metabolic block in cancer cells. (**a**) Viability assay performed with respiratory chain deficient cells (*ρ*^0^) and control (wild type) cells treated with HMGB1 (160 nM, 72 h, *n*=3). **P<0.0001 (*t*-test). (**b**) The amount of ATP production was calculated from O_2_ consumption and from ^13^C-lactate efflux derived from ^13^C-labelled glucose (Supplementary Fig. S2A). (**c**) The amount of ATP production was calculated from ^13^C-lactate efflux derived from ^13^C-labelled glutamine (Methods section). Cells were treated with HMGB1 (80 nM, 24 h, *n*=3, *P<0.02, *t*-test). (**d**) Survival of cells after treatment with oligomycin (10 ng ml^−1^) and HMGB1 (80 nM; both 72 h, *n*=3, **P<0.0001, *t*-test). (**e**) Crystal violet survival assay in glucose-free medium after treatment with HMGB1 (80 nM, SW480 and HCT116; 160 nM, HT29; 24 h). L-DON (1 μM) was added as indicated (*n*=3, *P<0.02, *t*-test). (**f**) After siRNA-mediated knockdown of ME1 the HT29 cells were treated with HMGB1 (80 nM, 72 h, *n*=3, *P<0.0003, **P<0.000002, *t*-test). (**g**) Immunoblot with anti-ME1 antibody to confirm the knockdown. Error bars represent s.d.

**Figure 8:**
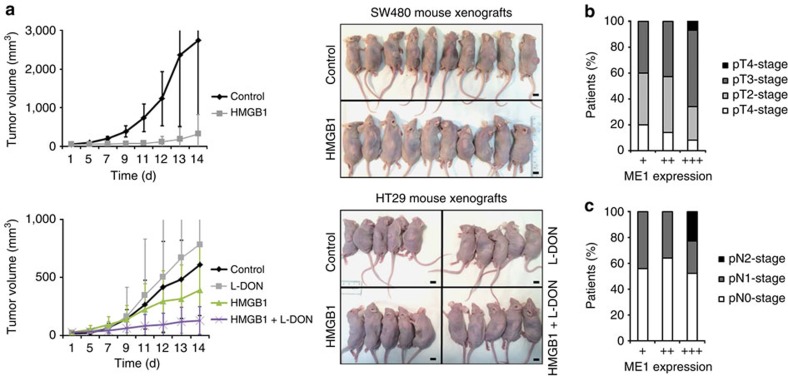
**HMGB1 kills CRC cells** ***in vivo***. (**a**) Systemic (intraperitoneal) HMGB1 treatment of CD1 nude mice bearing subcutaneous SW480 (*n*=20, *P*<0.05 (*t*-test) HMGB1 versus control) or HT29 (*n*=20; *P*<0.05 (*t*-test) for HMGB1+L-DON versus control or L-DON or HMGB1) xenograft tumours was started when tumours were palpable (time point 0). Control mice were injected with saline. The tumour volumes were determined at regular intervals. Scale bars, 1 cm. (**b**) ME1 expression levels and local invasion depth of cancer tissue (pT stage) in patients with rectal carcinoma;+low, ++ moderate, +++ strong expression of ME1. (**c**) Association of ME1 expression levels and lymph node metastasis (pN stage) in patients with rectal carcinoma. Error bars represent s.d.

The observed cell death induced by specific inhibition of the PK M2 tetramer and consequent inhibition of glucose-driven respiration should favour the survival of cancer cells performing mainly (anaerobic) glycolysis. To test this hypothesis we generated CRC cells devoid of an intact respiratory chain (*ρ*^0^ cells) from one HMGB1-sensitive (SW480) and one partly HMGB1-sensitive (HCT116) cell line. These modified cell lines, performing solely glycolysis, became almost completely resistant to HMGB1 (Fig. 7a). To assess the relative contributions of glycolysis, glutaminolysis and aerobic respiration to cellular survival in presence of HMGB1, we calculated total ATP generation (Fig. 7b,c) from the lactate production rates and from the oxygen consumption. Both HMGB1 partly (HCT116) and highly resistant (HT29) cancer cells compensated the HMGB1-caused decline of ATP production efficiently by glycolysis, whereas SW480 cells showed a strong decline of ATP production of ∼50% (Fig. 7b). However, after HMGB1 treatment, only HT29 cells could sustain ATP production from aerobic respiration by employing glutaminolysis, as ATP yield from glutaminolysis (Fig. 7c) was in good agreement with ATP produced by O_2_ utilization (Fig. 7b). Consistently, after HMGB1 treatment of HT29 cells glucose oxidation was strongly decreased (∼50%) and glutamine oxidation increased (∼35%) as assessed by measuring production of labelled CO_2_ (Supplementary Fig. 4B). Importantly, energy from aerobic respiration was critical for survival of SW480 and HCT116 cells as shown by induction of rapid cell death by oligomycin (Fig. 7d). Inhibition of glutaminolysis by 6-diazo-5-oxo-L-norleucine (L-DON) resulted in synergistic cytotoxicity in both glucose deprived (Fig. 7e) and glucose supplemented medium (Supplementary Fig. 4I). After downregulation of malic enzyme 1 (ME1) we observed sensitization of HT29 cells towards HMGB1 cytotoxicity (Fig. 7f,g, Supplementary Fig. 4J). Further, HMGB1 inhibited the growth of HMGB1-sensitive SW480 xenograft tumours in nude mice (Fig. 8a), whereas treatment with a combination therapy of HMGB1 and L-DON substantially inhibited the growth of HMGB1-resistant HT29 xenograft tumours (Fig. 8a). Taken together, both enhanced glucose fermentation and increased glutaminolysis might render cancer cells resistant to HMGB1 and animal experiments suggest that treatment with recombinant HMGB1 could represent a therapeutic option.

### ME1 as a biomarker for CRC

We reasoned that increased expression levels of ME1 in human colorectal carcinomas *in vivo* could be an indicator for the ability of the tumour to maintain optimal energy supply under varying glucose concentrations. Thus, we performed immunohistochemical stainings of ME1 using a tumour microarray (TMA) containing 1,260 CRC specimens from a large case–control study (DACHS (Darmkrebs: Chancen der Verhütung durch Screening) study, Supplementary Fig. 4D,F). Specificity of the antibody was confirmed via ME1 knockdown and overexpression experiments (Supplementary Fig. 4G,H). We found consistently significant dependencies between the pathological tumour stage (pT stage, that is, the extent of local invasion into the bowel wall) and expression of ME1 (linear by linear association test, *P*<0.001). Remarkably, for the subgroup of patients with rectal cancer (*n*=362) only individuals with strong ME1 expression (+++) had locally advanced tumours (classified as pT4, Fig. 8b) or advanced lymph node metastasis (classified as pN2, Fig. 8c), suggesting a link between high-ME1 expression levels and advanced, aggressive tumours.

Moreover, we performed immunohistochemical staining of endogenous HMGB1 in the same TMA collection of samples (DACHS study, Supplementary Fig. 4D,F). However, no statistically significant association between HMGB1 expression and patient survival was observed.

## Discussion

We recently described that recombinant human HMGB1 efficiently induces a novel form of cell death in tumour cells^9^. This cell death was independent of HMGB1 interaction with its receptors. Cytosolic granules of NK cells and other immune cells are one of the natural sources of the HMGB1 protein^2^. Thus, we examined whether HMGB1 mediates tumour cell death during an antitumour immune response. To our knowledge there have been no reports about direct cytotoxic effects of NK cell-derived HMGB1, although its active secretion from immune cells is well documented. Several reports have demonstrated that high amounts of HMGB1 are secreted from NK cells on activation of the natural cytotoxicity receptor Nkp30 (ref. 4). Recently, NK cell-mediated tumour cell lysis was reported in HT29 colon cancer cells^13^, but the responsible lytic mechanism was not identified. Using an inhibitor (glycyrrhizin) of HMGB1 that directly binds to the protein we could show that HMGB1 derived from NK cells from blood donors and from NK cell lines killed CRC cells. Thus, besides other known lytic molecules secreted by NK cells such as proteases-like granzymes and perforins, HMGB1 contributes to the cytotoxic effects of NK cells. Local concentrations of HMGB1 at the immunological synapse – the interface of effector and target cell – can even be higher than the concentrations in the supernatants used in our study. Further studies using co-culture cytotoxicity assays will have to address this question.

The SW480 cell line, which was a ‘HMGB1-responding' model in Fig. 1c, was not affected in Fig. 1d. However, in Fig. 1c we used purified (recombinant or HPLC isolated) HMGB1, whereas in Fig. 1d we used NK cell supernatants. Therefore, we hypothesize that in the latter experimental setting the cytotoxic effect of HMGB1 was masked by other lytic molecules secreted by NK cells such as proteases-like granzymes and perforins. Moreover, glycyrrhizin might not be able to inhibit HMGB1 activity with high specificity in a supernatant that contains various other lytic and potentially interfering compounds. To address these limitations by an alternative approach, we performed additional experiments after HPLC isolation of HMGB1 from two independent blood donors, followed by a modified protocol of the original experiment (Methods section). HPLC isolation of HMGB1 from Nkp30-stimulated supernatants and subsequent transfer to IgG1 control supernatant resulted in substantial cytotoxicity and confirmed that HMGB1 is an important factor that has the specific ability to kill cancer cells.

Under some circumstances HMGB1 can be related to autophagocytosis, however, we did not observe any autophagosomes on treatment with HMGB1 by electron microscopy. Importantly, characteristics of classical types of cell death such as apoptosis or necroptosis were not observed^9^. Regarding the HMGB1-induced formation of giant mitochondria, it is important that the reduction of mitochondrial respiration and perturbation of mitochondrial structure typically induce glucose fermentation (that is, the conversion of glucose to lactate, also called ‘anaerobic glycolysis'). A key enzyme responsible for channelling glucose flux to lactate and anabolic reactions is PK isoform M2 (PK M2). In colon carcinoma, PK M2 is the most abundant isoform of this protein existing in a dimeric and a tetrameric state. Dimeric PK M2 is a typical marker of fast proliferating non-malignant and cancer cells. It is weakly active at physiological phosphoenolpyruvate (PEP) levels and thereby redirects the gylcolytic flux into the pentose phosphate shunt^14^. The tetrameric PK M2 is characterized by a high Km for PEP (ref. 15) and supplies the mitochondria with pyruvate thus enabling aerobic respiration^14^. It is well-known, however poorly understood, that increased levels of PK M2 dimer result in increased lactate production.

We could show that both purified and NK cell secreted HMGB1 protein specifically inhibited the tetrameric form of PK M2. The glycolytic flux was decreased but not the enolase enzyme activity itself, confirming that HMGB1 specifically inhibited the PK M2 tetramer not directly affecting other glycolytic enzymes. The accumulation of glucose intermediates upstream of PK caused a strong increase in the hexokinase product glucose-6-phosphate that could well explain the observed decrease in hexokinase activity by product inhibition.

We observed strong binding of the PK M2 enzyme and HMGB1 *in vitro* and provided further evidence for this interaction in our *in silico* docking studies. HMGB1 contains two DNA-binding domains—the A box and the B box—and a highly acidic, C-terminal tail^16^. It is known that the HMGB1 protein which is actively secreted from immune cells, is polyphosphorylated^17^. PTyr peptide binding was shown to catalyse the release of FBP—a well-known allosteric activator of PK M2 (ref. 18)—from PK M2 and to result in inhibition of PK M2 activity. Similarly, phosphorylation of PK M2 (Y105) was reported to shift the dimer:tetramer equilibrium towards the (less active) dimer form by disrupting the binding of FBP to PK M2 (ref. 12). These reports are in line with our findings: first, we show that HMGB1 phosphorylated on its tyrosine residues binds in the vicinity of the allosteric binding site of FBP. Second, Y105 is in the vicinity (8 Å) of the proposed HMGB1 binding site, suggesting that HMGB1 and Y105 phosphorylation represent competitive inhibitory mechanisms. Thus, both binding of FBP to PK M2 and phosphorylation of PK M2 (Y105) compete with HMGB1 binding, arguing for HMGB1 being a novel allosteric inhibitor of tetrameric PK M2.

It is likely that also human HMGB1 protein produced in E. coli (used in several of the experiments in this study) is polyphosphorylated by the bacterial endokinases as shown for many other recombinant proteins^19^. Therefore, the amount of the phosphorylated HMGB1 fraction in the recombinant protein sample may influence its cytotoxicity.

It is likely that HMGB1 in excess—for example, during sepsis^1^—at least partly contributes to the failure of organs through inhibition of cellular glucose-driven respiration. Our results could also explain the observation that HMGB1 can function as an important antibiotic peptide^6^ by blocking the glycolytic activity of bacteria through inhibiting the PK enzyme that is highly conserved across species^20^. Thus, HMGB1 stored in secretory granules of NK cells could be an important cytotoxic compound of the innate immune system, affecting the energy metabolism of diverse pathogens and particularly of cancer cells through inhibition of tetrameric PK M2.

Because of the strong inhibition of aerobic respiration by HMGB1 in cancer cells with relatively high mitochondrial respiration (SW480, HCT116), we hypothesized that cancer cells adapted to anaerobic metabolism could be resistant to HMGB1. Thus we impaired their respiratory chain. In fact, after this modification they turned completely resistant to HMGB1 confirming that HMGB1 targets aerobic respiration by inhibition of tetrameric PK M2 and forces cells to rely on (anaerobic) glycolysis employing dimeric PK M2. Importantly, HMGB1 did not inhibit the uptake of glucose, rather it redirected glucose flux to anabolic (pentose phosphate shunt) reactions and lactate.

In addition to glucose, glutaminolysis (incomplete glutamine oxidation) can give rise to over 50% of cellular ATP from OXPHOS and maintain respiration^21^. HMGB1-resistant cells (HT29) performed *per se* high anaerobic glycolysis and could additionally employ glutaminolysis for mitochondrial ATP generation. Only this resistant cell line displayed a strong cytotoxic synergy between the oligomycin-induced inhibition of overall energy production from aerobic respiration and the HMGB1-induced block of energy production from glucose-driven aerobic respiration. Thus, the brisk inhibition of glucose-driven respiration by HMGB1 was best tolerated by cancer cells that could efficiently upregulate glucose fermentation and maintain aerobic respiration by glutamine break-down. To target this rescue mechanism we performed a combined treatment with low, non-cytotoxic doses of the glutamine-analogue L-DON and HMGB1 in glucose deprived and high glucose media, respectively. This treatment resulted in a sensitization of the cells to HMGB1 with a stronger effect under glucose deprived cell culture conditions. Consistently, in nude mice bearing HMGB1-resistant HT29 xenograft tumours, treatment with non-cytotoxic L-DON doses and HMGB1 resulted in a sensitization of the xenografts to HMGB1.

As relatively high glutaminolytic rates yielding glutamine-derived lactate (Fig. 7c) defined HMGB1-resistant CRC cells in our model, we focused on the ME1 that enables the final conversion of glutamine to lactate by catalysing the oxidation of malate to pyruvate. Knockdown of ME1 in the HMGB1-resistant colorectal cell line HT29 disabled the glutaminolysis-dependent HMGB1 resistance. There are only few reports on malic enzyme activity in cancer cells^22^. In our model, CRC cells were killed due to HMGB1-mediated dissociation of glycolysis and mitochondrial respiration. Glutaminolysis could rescue the cells by maintaining sufficient respiration and energy production, providing cells with highly active ME1 a survival advantage over those with less active ME1. The association between ME1 expression and tumour stage in a human CRC TMA comprising 1,260 specimens was in line with our results demonstrating the importance of glutaminolysis for CRC growth. However, ME1 (and HMGB1) expression did not correlate with survival in our study (Supplementary Fig. S4E and data not shown) suggesting that local tumour growth and lymphatic spreading but not the formation of distant metastasis are driven by glutaminolysis. We hypothesize that ME1 levels might be elevated during loco-regional spread of tumour cells when tumour cells still need a basal aerobic respiration. In the process of metastatic spread they switch to anaerobic glycolysis adapting to scarce nutrients and hypoxic/anoxic microenvironment. These metastatic cells are most indicative of the prognosis and survival in CRC and might not rely on preservation of basal respiration via ME1.

There are ongoing efforts to implement therapies targeting glutaminolysis in the treatment of human cancer^23^. Assuming that HMGB1 is a functionally important cytotoxic compound of the innate immune system, targeting glutaminolysis could enhance the immune system's response against cancer. Moreover, application of recombinant HMGB1 would potentiate this natural cytotoxicity. Finally, glucose withdrawal might potentiate the inhibitory effects of these compounds by substrate reduction. However, the potential cytotoxic side effects of HMGB1 have to be carefully evaluated in appropriate pre-clinical and clinical trials.

## Methods

### Cell culture and animal studies

Human colon carcinoma cell lines SW480, HCT116, HT29 and Caco2, the human glioblastoma cell line U251MG and the NK cell line NK-92 Cl were purchased from ATCC. Cell lines were regularly tested for contamination by multiplex PCR performed in the Genomics and Proteomics Core Facility^24^ (DKFZ, Heidelberg, Germany). The cell lines used in the study are not on the ICLAC and NCBI biosample list of misidentified cell lines^25^. For experiments, cells were cultured for no more than 10 passages. All cell lines were tested for mycoplasma contamination at regular intervals; there was no sample contamination with mycoplasma. Human NK cells were purified out of leukocyte concentrates. Cells used in the experiments were cultured in Roswell Park Memorial Institute medium (RPMI; #1640, colon carcinomas, NK cells) or DMEM high glucose (#41965-039, glioblastoma cells) medium. Rho zero cells were generated as described earlier^9^. Briefly, cells were cultured in RPMI medium (10% foetal bovine serum, 1% P/S) supplemented with 250 ng ml^−1^ ethidiumbromide, 50 μg ml^−1^ L-pyruvate and 5 mg ml^−1^ uridine over a period of 12 weeks. For cytotoxicity measurements, cells were cultured in 96-well plates, treated with recombinant human HMGB1 protein (Sigma-Aldrich), glycyrrhizinic acid ((3β, 18α)-30-hydroxy-11, 30-dioxoolean-12-en-3-yl 2-O-β-D-glucopyranuronosyl-β-D-glucopyranosiduronic acid); Sigma-Aldrich), 10 μM (non-toxic) or 100 μM (cytotoxic) P-M2tide (aa sequence: GGAVDDDpYAQFANGG; #BML-P239-0001; Enzo Life Sciences) or 100 μM ML-265 (Cayman Chemical), then cell viability was assessed by crystal violet staining^26^. ME1 knockdown was performed with 40 nM siRNA using lipofectamine in 6-well plates followed by treatment with 80 nM HMGB1 for 72 h. Sequences of siRNA were: ME1, 5′-CCCUGUGGGUAAAUUGGCUCUAUAU-3′ and scrambled control 5′-CCUGCAGUACUUCAAGCGGtt-3′. PKM2 siRNA was from Santa Cruz. A non-specific siRNA served as control (Dharmacon, Schwerte, Germany). For overexpression of PKM2 or ME1, cells were transfected with pCMV-PKM2 (Sino Biological Inc., Beijing, China) or pCMV-ME1 (OriGene, Rockville, MD, USA) using Lipofectamine 2000. For cytotoxicity measurements confluent cells were cultured in 96-well plates if not otherwise indicated. Cytotoxic activity of supernatants from stimulated NK cells was assessed in 96-well plates for 3 days with RPMI medium as reference. To avoid unspecific cytotoxicity in the IgG1 control we diluted the supernatant derived from the NK cells from the blood donors until no substantial cell death occurred in the IgG1 control group. Next, we diluted the Nkp30-stimulated supernatants using the same dilution factor. Consistently, IgG1 controls showed no cytotoxicity, whereas Nkp30 supernatants were cytotoxic. Further, we added HMGB1 isolated from the Nkp30 supernatant by HPLC to the non-toxic IgG1 supernatants which resulted in the induction of substantial cell death.

*Animal studies*. For animal studies six-week-old female and male athymic CD1 nude mice (Charles River, *n*=40) were injected subcutaneously with 5 × 10^6^ SW480 or HT29 cells in 100 μl PBS in the right flank using a 30-gauge needle. Treatment was started when tumours were palpable. Daily intraperitoneal injections at the contralateral side for 2 weeks were done with 10 μg rhHMGB1 in 500 μl PBS or PBS only (control group) and/or 12.5 mg kg^−1^ per injection L-DON (ref. 27). Tumour volume was measured by a calliper using the ellipsoid formula (length × width × height × ½) as described^28^. After 2 weeks of treatment the animals were killed. All animal work was carried out in accordance with the NIH guidelines Guide for the Care and Use of Laboratory Animals. This study was approved by the institutional review board of Heidelberg University Hospital.

### Reversed-phase HPLC purification and identification of HMGB1

Reversed-phase chromatography: HMGB1 was extracted and purified by reversed-phase chromatography referring to Zetterström *et al.*^6^ with the exception that Source 15 media were applied for chromatography. For the first purification step a Resource RPC column (6.4 × 100 mm; GE Healthcare) was applied. Solvent A was water with 0.17% trifluoroacetic acid (TFA), solvent B was acetonitrile with 0.15% TFA. Flow rate was 1 ml min^−1^. The following elution programme was performed: 5% solvent B isocratic for 10 min, 5–30% B linear for 15 min, 30–60% B linear for 45 min, 60–90% B for 5 min and 90% B isocratic for 5 min. The second purification step was conducted on a Source 15RPC ST 4.6/100 column applying the same elution conditions as described above. Final purification was achieved on the Source 15RPC ST 4.6/100 column by elution with 5% B isocratic for 10 min, 5–40% B linear for 15 min, 40–50% B linear for 45 min, 50–90% B for 5 min and 90% B isocratic for 5 min.

NK-92 CI cells were cultured in minimum essential medium alpha (Gibco) supplemented with 12.5% foetal bovine serum (Gibco), 12.5% horse serum (Life technologies GmbH), 0.1% 2-mercaptoethanol (Gibco) and 100 IU ml^−1^ penicillin and 100 μg ml^−1^ streptomycin (both Sigma-Aldrich). Cells were split and expanded by carefully rocking the culture flasks on a daily basis and adding fresh medium on necessity. Twenty-four hours before harvesting the cells, recombinant human interleukin-2 (IL-2; Tecin from Roche, kindly provided by the NIH) was added to a concentration of 100 IU ml^−1^. In all, 6 × 10^8^ NK-92 CI cells were harvested from 1.8 l of culture medium and used for purification of intracellular membraneous vesicles as described^29^. Coomassie blue staining of all eluates (80) was performed with Brilliant Blue R-250 dye (Sigma) according to standard protocols. HMGB1 was detected by immunoblot analysis using human anti-HMGB1 antibody (1:1,000, abcam). Silver staining: the gel was stained with the Pierce Silver Stain Kit (Thermo Scientific, Rockford, IL) according to the manufacturer's instructions.

### Preparation and culture of human NK cells

Human NK cells were purified out of leukocyte concentrates from the blood bank in Mannheim (Germany). The use was approved by the local ethics committee of the University of Heidelberg and the medical boards of Baden–Wuerttemberg and Rhineland-Palatinate. Written informed consent was obtained from each participant. The concentrate was diluted with PBS and subjected to a centrifugation step on biocoll separation solution (Biochrom AG). The buffy coat was harvested and plastic adherence was carried out for 45 min. Out of the obtained peripheral blood leukocytes NK cells were isolated with the human NK cell isolation kit (Miltenyi) according to the manufacturer's instructions. Highly pure NK cells (95% CD3- CD56+ cells as determined by flow cytometry) were then cultured in CellGro stem cell growth medium (CellGenix) with 10% human AB serum (PAA Laboratories), 200 U ml^−1^ recombinant human IL-2 (National Institutes of Health) and 100 U ml^−1^ penicillin and 100 mg ml^−1^ streptomycin (Sigma-Aldrich) at a density of 1 × 10^6^ cells ml^−1^. After 6 days the NK cells were harvested, counted and re-seeded at a density of 2 × 10^6^ cells ml^−1^ in antibody pre-coated wells of a 96-well plate in RPMI (Sigma-Aldrich) supplemented with 10% foetal calf serum (Invitrogen) and 100 U ml^−1^ IL-2. For the coating, 1 day before seeding the cells, the wells were incubated with 1 μg ml^−1^ of either mIgG1 (clone MOPC-21) or anti-NKp30 antibody (clone P30-15, both from BioLegend) in PBS overnight at 4 °C. After 2 days on the pre-coated plates, the supernatants were harvested and centrifuged to pellet potential cellular contaminants. Aliquots of the supernatants were used for performing an IFN-γ ELISA (BioLegend) according to the instructions provided by the manufacturer.

### *Ex vivo* colon carcinoma specimens and tissue microarray

Immediately after the surgical removal of the colon part containing the tumour, a fresh tumour biopsy was processed with a vibrating blade microtome (Vibratome, Leica). Tissue slices of 300 μm were generated and incubated for the indicated times in RPMI cell culture medium. Control sections were fixed overnight in buffered 4% formalin (pH 7.4) solution, then paraffin embedded and haematoxylin and eosin staining was performed on an automated staining system (Techmate 500, DakoCytomation). Haematoxylin and eosin-sections were reviewed by pathologists (WR, GG) for the presence of colorectal carcinoma. All surgical specimens were obtained from the Department of General, Visceral and Accident Surgery of the Heidelberg University Hospital (Germany). The use of the human tissue for study purposes was approved by the local ethics committee at the Heidelberg University Hospital. Written informed consent was obtained from each participant at baseline, including the assignment of tumour tissue from patients with CRC.

For creation of the TMA, tissue samples from 1.260 colorectal carcinoma patients, included in the German DACHS (Colon Cancer: Chances of Prevention through Screening) case–control study^30^, were collected by the tumour Tissue Bank of the National Center for tumour Diseases in Heidelberg. The use of the human tissue was approved by the local ethics committee of the University of Heidelberg and the medical boards of Baden–Wuerttemberg and Rhineland–Palatinate. Written informed consent was obtained from each participant at baseline, including the assignment of tumour tissue from patients with CRC.

The DACHS study is an ongoing population-based case–control study located in southwest Germany with comprehensive assessment of clinical and epidemiological data. Patients with a histologically confirmed first CRC diagnosis with their first residence in the study region were eligible for this study if they were at least 30 years old, physically and mentally able to participate, and able to speak German. The patients included in this analysis were recruited between 2003 and 2007 in all 22 hospitals of the study region offering CRC surgery. The patients gave information during a face-to-face interview conducted by a trained interviewer. The standardized questionnaire included questions on sociodemographic status, lifestyle and reproductive factors, as well as the family history and medical history of the patients. In addition, discharge letters and pathology reports and, if applicable, endoscopy reports of previous colorectal endoscopies were collected. This part of the study was covered by the ethical approval mentioned above.

Formalin-fixed paraffin-embedded surgical specimens were requested from the pathology departments of the cooperating clinics and transferred to the tissue bank of the National Center for tumour Diseases in Heidelberg, where tumour tissue was incorporated into tissue microarray blocks. Written informed consent was obtained from every patient, including the assignment of tumour tissue from patients with CRC.

On average 3 years after diagnosis, a questionnaire was sent to the treating physicians of the patients to collect information on CRC therapy, newly diagnosed concomitant diseases and recurrences of CRC (completeness of records: 99%). Data on vital status and date of death were obtained from the population registries and the cause of death was verified by death certificates obtained from the health authorities in the Rhein–Neckar–Odenwald region (completeness: >99%). New diagnoses and cancer recurrences were verified through medical records of the attending physicians. For patients without any event of interest, censoring occurred at the date of last follow-up or 31 December 2009, whichever came first.

### Immunohistochemistry

TMA sections were immunostained as described earlier^31^ using an automated staining system (Techmate 500, DakoCytomation). Visualization was done with avidin–biotin-complex peroxidase, aminoethylcarbazole and haematoxylin. The sections were incubated with the rabbit polyclonal anti-ME1 antibody (1:100, ab97445, abcam) and processed with the following kits: ChemMate Detection Kit (K5003, DakoCytomation), ChemMate Buffer Kit (K5006, DakoCytomation) and Avidin/Biotin Blocking Kit (SP-2001, Vector Laboratories). A product of the scores of staining intensity and quantity of positive cancer cells was assessed semiquantitatively and independently by two pathologists (W.R. and G.G.). Herein the intensity range was 0=negative; 1=low; 2=medium and 3=high and the quantity 0=no positivity; 1=positivity in 0–10%; 2=positivity in 11–50%; 3=positivity in 51–80%; 4=positivity in >80%. For few cases of discrepant validation a consensus score was determined. The staining and evaluation was additionally performed on a second TMA giving similar results. The final immunoreactive score (IRS, ranging from 0 to 12) is obtained by multiplication of the intensity score and the quantity score. For ME1 low, moderate and strong positive expression was defined as IRS<3, IRS between 3 and 6, and IRS>6, respectively. For HMGB1 low and high expression was defined as IRS between 1 and 6, and IRS>6, respectively. ME1 antibody specifity: cells were plated and transfected on glass coverslips in 6-well plates. The coverslips were collected, fixed with paraformaldehyde and immunostained with ME1 antibody as described for the TMA sections.

### Electron microscopy

Electron microscopy was performed as previously described^9^. Cells were fixed (2.3% glutaraldehyde in 50 mM sodium cacodylate, pH 7.2) *in situ* for 30 min at 4 °C, scraped, centrifuged at 200*g* for 10 min at 4 °C and stained (2% osmium tetroxide and 5% uranyl acetate). Ultrathin sections from dehydrated and Epon embedded samples were microphotographed with a Zeiss EM-10A electron microscope at 80 kV. Grating replica suited as controls for the magnification indicator.

### Enzymatic assays

Enzymatic activities of respiratory chain complexes, glycolytic proteins and malic enzyme were determined in subcellular fractions as previously described^32^,^33^ using a computer-tuneable spectrophotometer (Spectramax Plus Microplate Reader, Molecular Devices; Sunny Vale, CA, USA) operating in the dual wavelength mode; samples were analysed in temperature-controlled 96-well plates in a final volume of 300 μl. Activity of ME1 was recorded in presence of increasing amounts of malic acid (0.02, 0.05, 0.1, 0.2, 0.5, 1 and 2.5 mM). *V*_max_ and *K*_m_ were calculated using a Hanes–Woolf plot. In the presence of high substrate levels the *K*_m_ for malic acid was similar in all three tested cell lines (SW480: 0.32 mM, HCT116: 0.30 mM, HT29: 0.31 mM). *V*_max_ (mU mg^−1^ protein) values were 3.38 (SW480, 0.5–5.0 mM malic acid), 5.77 (HCT116, 0.5–5.0 mM malic acid) and 3.68 (HT29, 0.5–5.0 mM malic acid) and 1.68 (SW480, 0.02–0.2 mM malic acid), 3.24 (HCT116, 0.02–0.2 mM malic acid) and 1.89 (HT29, 0.02–0.2 mM malic acid). Two isoforms of ME1, mitochondrial (NAD(P)+dependent) ME3 and mitochondrial (NAD+ dependent) ME2, had very low or no detectable activities (data not shown). Dimeric PK M2 is virtually inactive at physiological PEP levels allowing differentiation of both forms by using very high (10 mM) and low (100 μM) amounts of PEP in the enzymatic assay.

Glucose-6-phosphate levels in cells were measured using the Glucose-6-phosphate assay kit (Sigma) according to the manufacturer's protocol.

### Metabolic assays

Mitochondrial respiratory rate was measured according to a previously described protocol^34^ using computer-supported high-resolution Oroboros 1 oxygraph system (Paar, Graz, Austria). Cells were grown to confluency in 6-cm plates and treated with HMGB1 (80 nM, 24 h) if indicated. For *ex vivo* tissue slices two slices per chamber were used and, if indicated, treated with 160 nM HMGB1 for 24 h. Cellular respiration was measured in RPMI medium. Mitochondrial respiration (‘delta PO_2_') was calculated by subtracting background oxygen consumption rate (that is, NaCN (5 mM) insensitive respiration) from total oxygen consumption rate. Glycolysis was measured by monitoring the conversion of 5-^3^H-Glucose to ^3^H_2_O as described by Liang *et al.*^35^ Briefly, following trypsinization cells were washed in PBS and resuspended in 1 ml Krebs buffer containing 10 mM glucose, and spiked with 370 MBq 5-^3^H-Glucose (Hartmann Analytic, Braunschweig, Germany). Following incubation for 1 h at 37 °C diffusion through a PCR vial was used to separate ^3^H_2_O formed by glycolysis. Radioactivity was determined in a liquid scintillation counter (TRICARB 2900, PerkinElmer).

Incorporation of ^14^C into RNA ribose from U-^14^C-labelled glucose (Hartmann Analytic) was taken as a measure of glucose utilization in the pentose phosphate pathway^36^. To this end 370 MBq ^14^C-glucose was included in the culture medium for 24 h. Subsequently RNA was isolated applying a commercial kit (Qiagen) and ^14^C-incorporation was quantitated by liquid scintillation counting.

To measure CO_2_ production in the presence of labelled glucose and glutamine, respectively, 5 μCi L-[^14^C(U)]-glutamine (74 GBq mmol^−1^) or 5 μCi D-[(^14^C(U)]-glucose were added to the cell cultures grown (HT29 cells) with NaHCO_3_-free medium in 6-well plates. The cells incubated for 1 h. To trap released ^14^CO_2_ the lid of the well was equipped with a filter paper soaked with Hyamine. The wells were gas tight sealed with parafilm. At the end of the incubation 500 μl 10% trichloroacetic acid (TCA) were injected through the lid to expel ^14^CO_2_ dissolved in the medium. Finally the filter papers were transferred to LSC vials mixed with 10 ml Ultima Gold LSC cocktail and counted for radioactivity^37^.

The calculation of total ATP production from aerobic respiration and glycolysis was done assuming that during aerobic respiration 6 ATP molecules are produced per O_2_ molecule consumed and that one ^13^C-lactate molecule (from glucose) yields one ATP molecule^38^,^39^. The calculation of contribution of glutaminolysis to ATP production was done assuming that during oxidative phosphorylation 2.5 or 1.5 ATP were produced per NADH or FADH molecule, respectively, and that one ^13^C-lactate molecule (from glutamine) yields one GTP/ATP molecule from substrate level phosphorylation^40^.

For the ^125^I experiments (immunoprecipitation and cell permeability towards HMGB1) radioactivity measurements cells were solubilised in 0.5 M NaOH and mixed with 10 ml LSC cocktail. Radioactivity was measured in a liquid scintillation counter as described earlier^9^.

### *In silico* HMGB1—PK M2 protein docking studies

The simulation of diffusional association (SDA) programme^41^ was used to simulate the diffusional encounter of monomeric PK M2 (Protein Data Bank (PDB): 3BJF) with both the phosphorylated and unphosphorylated HMGB1 box A and HMGB1 box B. The individual HMGB1 box domains (PDB: 1CKT, 2YRQ) were used rather than the complete HMGB1 structure due to the complexity of accurately accounting for the structural flexibility of the linker region (residues 79–94) between the two domains. Initial structures were taken from the PDB, and X-ray structures were taken preferentially over nuclear magnetic resonance (NMR) structures where possible. The PK M2 structure (PDB code: 3BJF), HMGB box A (PDB code: 1CKT), HMGB box B (PDB code: 2YRQ, residues 95–163) were used. All calculations used the chain A from 3BJF. For the calculation with FBP present, this residue was saved as a mol2 file in UCSF Chimera^42^, and submitted to the PDB2PQR web server^43^ in addition to the modified PDB file. In all other calculations, all ligands were removed from the structures. The PDB2PQR web server was used to prepare all structures for simulation with SDA, using the AMBER force field parameters, and protonation states assigned at pH 7. Each HMGB1 structure, and the PK M2 phosphorylated at Y105, was phosphorylated using the build feature of Chimera. Charges and radii were manually added to the PQR files using the phosphotyrosine parameters of Homeyer *et al.*^44^ Adaptive Poisson-Boltzmann Solver (APBS) version 1.2.1 (ref. 45) was used to solve the linearized Poisson–Boltzmann equation with simple Debye–Hückel boundary conditions, a protein dielectric constant of 1, and a solute dielectric of 78 to calculate the electrostatic potential for each protein on cubic grids of 129 points, with 1 Å grid spacing. The potential was calculated at 50 mM ionic strength, with positive and negative ions with 1.5 Å radius. Dielectric and ion-accessibility coefficients were calculated using the smoothed method (smol option), and the smoothing window was set to 0.3 Å. For the purposes of the effective charge calculations, test charges were placed as per the original SDA calculations, and additionally on the phosphorus and oxygen atoms of the phosphotyrosine residue. The SDA programme was used to calculate in excess of 40,000 docked encounter complexes of each of the four HMGB1 models (unphosphorylated Box A, phosphorylated Box A, unphosphorylated Box B, phosphorylated Box B) with PK M2. The SDA calculations included electrostatic interaction, electrostatic desolvation and hydrophobic desolvation terms with weighting factors 0.5, 1.0 and −0.013, respectively. The protein probe radius was set to 1.77 Å, solvent probe to 1.4 Å and an exclusion grid spacing of 0.5 Å. Proteins were initially separated by 260 Å, and a simulation was stopped if the centre–centre distance exceeded 540 Å or the total simulation time exceeded 5,000 ps. The top 5,000 docked complexes, as ranked by favourable interaction energy, were retained for cluster analysis using the hierarchical clustering tool provided with SDA. For each simulation, the docked complexes were clustered to produce 10 clusters for quantitative and visual analysis. All images were prepared using the VMD visualization software^46^.

### NMR analysis of metabolites

For mass isotopomer assays, cells were cultured in glucose- or glutamine-free medium supplemented with either uniformly labelled (U)-^13^C-D-glucose or U-^13^C-glutamine (Sigma-Aldrich). For analysis of ^13^C-lactate efflux 1 ml of the cellular supernatant was centrifuged (8,000*g*, 10 min, 4 °C) to spin down cellular debris. To 500 μl of the supernatant 10% of D_2_O were added respectively and transferred to 5 mm NMR sample tubes. Lactate derived from the metabolism of ^13^C_6_-D-glucose or ^13^C_5_-glutamine was determined by comparing the CH_3_ group intensities of labelled and non-labelled lactate in NMR. The samples were measured with a Bruker AvanceIII 600 NMR spectrometer, equipped with a cryogenically cooled detection probe (QNP-CryoProbe).

Parameters for measurement included:Magnetic Field 14.09 Tesla; sample temperature 295 K; pulse width 4.7 us (corresponding to 30° flip angle); Broadband Composite Pulse Decoupling (Waltz65) during acquisition and relaxation delay, 128 K total acquisition data points; acquisition time 1.8 s; relaxation delay 1.5 s; 512 transients; total experiment time 30 min.

*Processing parameters*. The processing parameters included: zero filling to 256 K real data points, exponential multiplication (lb=1.0 Hz); fourier transformation with backward linear prediction in order to compensate for baseline artifacts.

For data analysis, the integral of the signal of the ^13^CH_3_ group of lactate (singlet at *δ*=20.108 p.p.m. for non-labelled lactate and doublet for labelled lactate at *δ*=20.097 p.p.m. (^1^J(^13^C^13^C)=36.8 Hz) respectively) was taken as the measure of lactate concentration. To get reliable quantitative results, the intensities were calibrated with standard samples containing known amounts of labelled and non-labelled lactate. This procedure also compensates errors due to incomplete relaxation of the ^13^C nuclei within the chosen repetition time (3.3 s) The determination of concentrations was performed by using the ‘ERETIC' functionality built in the Bruker NMR software (Topspin 3.2, Bruker BioSpin 2012). The concentrations obtained in this way were corrected for the incomplete degree of ^13^C enrichments in ^13^C_6_-glucose and ^13^C_5_-glutamine respectively (98%).

### Immunoblot analysis and protein preparationm

Immunoblotting was performed according to standard procedures by SDS–polyacrylamide gel electrophoresis. Cells were lysed in lysis buffer P (20 mM Tris-HCl (pH 7.4), 137 mM NaCl, 10% (v/v) glycerine, 1% Triton X-100, 2 mM EDTA, 100 mM phenylmethylsulfonyl fluoride and protease inhibitors (Complete mini from Roche). Lysates were centrifuged at 14,000*g* (10 min) at 4 °C. Total protein was measured by the Bradford (Bio-Rad) method. Soluble protein was resolved by SDS–polyacrylamide gel electrophoresis, blotted onto nitrocellulose and incubated with one of the following antibodies: rabbit polyclonal anti HMGB1 (1:1,000, abcam, ab18256), mouse monoclonal anti GAPDH (1:1,000, Santa Cruz, sc-365062), mouse monoclonal anti-ß-actin (1:3,000, Sigma-Aldrich, A5441), rabbit anti PK M2 (1:1,000, Cell Signaling, #4053S), rabbit anti-ME1 (1:1,000, abcam, ab97445). Appropriate secondary antibodies (1:3,000, horse-radish peroxidase-conjugated, #170-6515 (goat anti rabbit IgG) and #170-6516 (goat anti mouse IgG)) were from Bio-Rad. Visualization was done by enhanced chemiluminescence technique (GE-Healthcare). Mitochondrial fractions were extracted using the ApoAlert Cell Fractionation Kit (Clontech) as described earlier^42^.

Binding of HMGB1 to PK M2 was tested by ultrafiltration of the PK M2-HMGB1 complex. Ultrafiltration of the PK M2-HMGB1 complex: equimolar amounts of HMGB1 and PKM2 were mixed in a final volume of 300 μl and filtrated (14000, g, 4 °C) to a final volume of 15 μl in an Amicon Ultra 0.5 ml 30 k device (Merck-Millipore, Darmstadt, Germany). The retentate was adjusted to the original volume after centrifugation. Then filtrate and retentate were analysed by Western Blot. For controls HMGB1 and PKM2 were also analysed alone. Pure HMGB1 (2 μM) suited as a negative control.

Uncropped scans of the most important western blots are supplied as Supplementary Figs in the Supplementary Information.

### *ME1* gene sequencing

Mutational analysis for U251MG, SW480, HCT116 and HT29 cell lines was performed by bidirectional Sanger-Sequencing of five overlapping complementary DNA fragments encompassing the complete coding region of *ME1*. The following primers were used for standard PCR amplification and direct sequencing on an ABI 3500 Genetic Analyzer (Life Technologies, Darmstadt, Germany): frag. 1 (forward) 5′-TGC AGT CAG CAC CGT CAC-3′, (reverse) 5′-CCT CGA TCG TGG ATA GTA A-3′; frag. 2 (forward) 5′-GGT CTG GCT TGC CAA CAA-3′, (reverse) 5′-ATC ATT GAA TGT GCA ATA CTG-3′; frag. 3 (forward) 5′-GAT TTT GCC AAT GTG AAT GC-3′, (reverse) 5′-GTT CTG AGA ATG CAC CAC CAA-3′; frag. 4 (forward) 5′-TGA AGA ACC TAG AAG CCA TTG-3′, (reverse) 5′-CTT GGT ATG CAT CTT TCA CAA TC-3′; frag. 5 (forward) 5′-GGA AGA GGG TCG GCT TTA T-3′, (reverse) 5′-ACC AGA AGG CAA AGT GAA GC-3′.

### Quantitative PCR analysis

Quantitative PCR analysis was performed as described previously^43^. The following primer pairs were used: 18S: 5′-CATGGCCGTTCTTAGTTGGT-3′ (forward), 5′-ATGCCAGAGTCTCGTTCGTT-3′ (reverse), ELF2: 5′-AGCTCCAAGGACAGTTCGTG-3′ (forward), 5′-GAGAGGTCGCTGTTGTTGGA-3′ (reverse), ELF4: 5′-CTGGAGTTGGACGACGTTCA-3′ (forward), 5′-AAGACTTCCGCGGTTGACAT-3′ (reverse), GAPDH: 5′-TCAAGAAGGTGGTGAAGCAG-3′ (forward), 5′-GGGTCTACATGGCAACTGTG-3′ (reverse), GLUT3: 5′-ATCTTCACCGGCTTCCTCAT-3′ (forward), 5′-GCTCGATGCTGTTCATCTCC-3′ (reverse), HMGB1: 5′-GGCCTTCTTCCTCTTCTGCT-3′ (forward), 5′-GCAACATCACCAATGGACAG-3′ (reverse), Oct3/4: 5′-CACTGCACTGTACTCCTCGG-3′ (forward), 5′-CTTTCCCTAGCTCCTCCCCT-3′ (reverse), p53: 5′-CACACCCTGGAGGATTTCAT-3′ (forward), 5′-AAGCGAGACCCAGTCTCAAA-3′ (reverse), PKM2: 5′-GGAAGTGGGCAGCAAGATCT-3′ (forward), 5′-AGGAAGTCGGCACCTTTCTG-3′ (reverse).

### Statistical analysis

We evaluated the association between ME1 or HMGB1 expression and local tumour extent (pT) and lymph node metastasis (pN) for all colorectal samples together as well as for the colon and rectal cancer subgroups using the linear by linear association test (Agresti A. Categorical Data Analysis. John Wiley & Sons. Hoboken, New Jersey, 2002). Overall survival time was defined as the time from diagnosis until death from any cause. End points for progression-free survival were tumour recurrence, distant metastases or death from any cause, whatever occurred first. For the analysis of CRC-related survival, deaths from unrelated causes were treated as competing events. Multivariate (cause-specific) proportional hazards regression models included ME1 or HMGB1 expression (IRS score), age, sex, grade, pT, pN, pM, tumour site, adjuvant and neoadjuvant chemo- and radiotherapy. The pT stadium is defined by the extent of tumour invasion into the colonic wall: submucosa (pT1), muscularis propria (pT2), subserosa/pericolic fat tissue (pT3) and perforation through peritoneum/invasion into other organs (pT4). The pN stadium is definied by the number of regional lymph node metastasis: metastasis in 1 regional lymph node (pN1a), metastasis in 2–3 regional lymph nodes (pN1b), tumour deposit(s) in the subserosa, or in the non-peritonealized pericolic or perirectal soft tissue without regional lymph node metastasis (pN1c), metastasis in 4 or more regional lymph nodes (pN2). The pM0 or pM1 stadium is definied by the absence or the occurrence of distant metastasis, respectively.

Results of laboratory experiments were analysed using paired *t*-tests. Results were illustrated using means±s.d. For all statistical tests a significance level of 5% was used (shown by asterisks). Significance in figures is shown by asterisks. Statistical analyses were performed using the statistical software environment R, version 2.15.3 and Microsoft Excel 2010 software.

## Additional information

**How to cite this article:** Gdynia, G. *et al.* The HMGB1 protein induces a metabolic type of tumour cell death by blocking aerobic respiration. *Nat. Commun.* 7:10764 doi: 10.1038/ncomms10764 (2016).

## Supplementary Material

Supplementary Information Supplementary Figures 1-4 and Supplementary References.
