# Supplementary material for: The HMGB1 protein induces a metabolic type of tumour cell death by blocking aerobic respiration
Source: Nat Commun. 2016 Mar 7;7:10764. doi: 10.1038/ncomms10764 (PMC4786644; doi:10.1038/ncomms10764)
Supplement: Supplementary Information — Supplementary Figures 1-4 and Supplementary References. [file ncomms10764-s1.pdf]

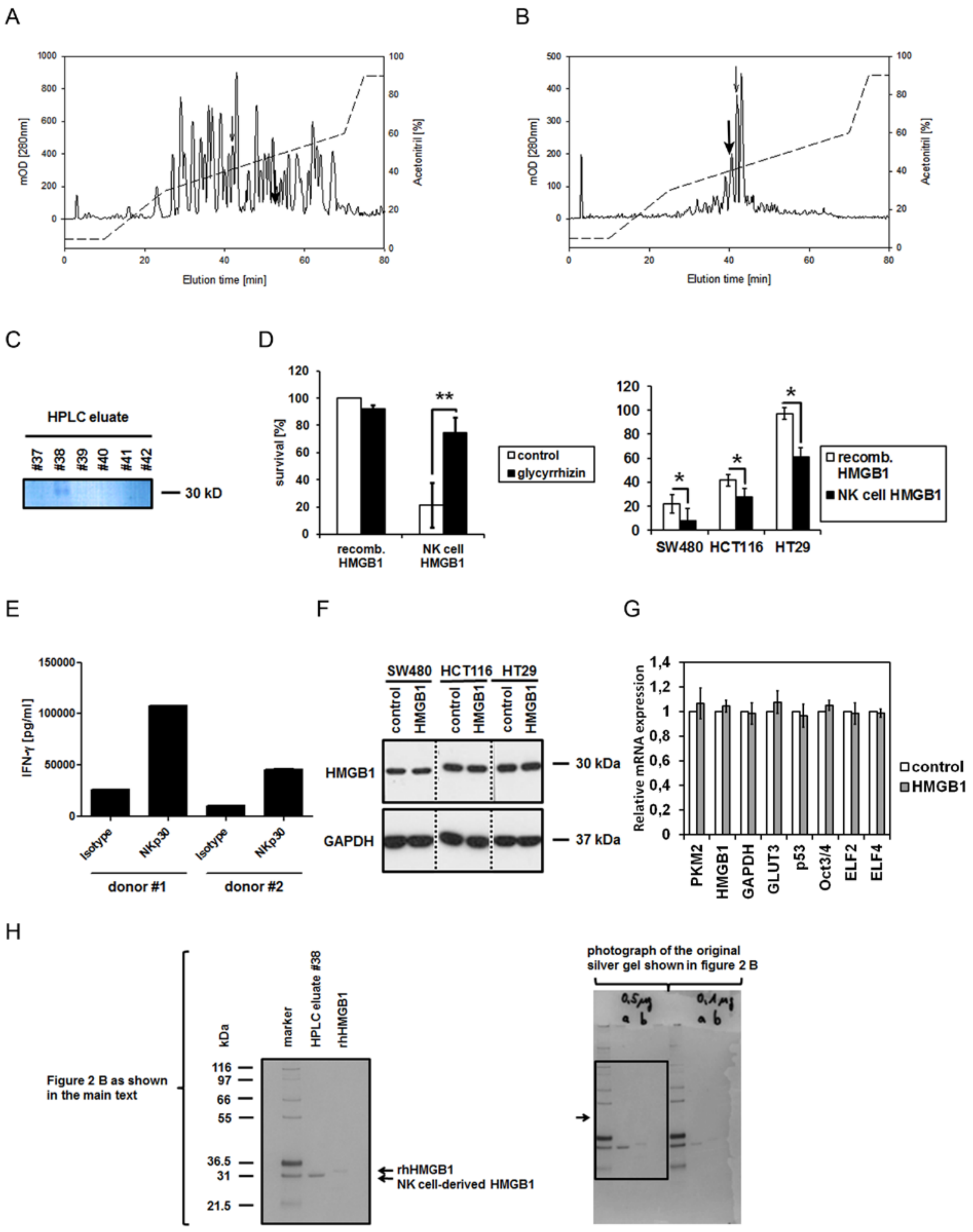

Supplementary Figure 1

NK cell derived HMGB1 induces cell death in colorectal cancer. (A, B) HMGB1 from cytotoxic granules from NK-92 CI cell line was purified by reversed phase chromatography on a Resource RPC

column (A) and on a Source 15RPC ST 4.6/100 column (B) before the final purification step. Details are given in the Experimental Procedures section. The dashed line indicates the acetonitrile gradient. HMGB1 containing fractions are indicated by arrows. (C) The purification yield was approx. 90% as determined by Commassie Blue staining of the corresponding gel. (D, left) Survival of HT29 cancer cells as assessed by crystal violet viability assay. Cells were treated with recombinant human HMGB1 (160 nM) or NK cell derived HMGB1 (160 nM); where indicated 200 µM glycyrrhizin was used (72h, n=3). (D, right) Side-by-side comparison of HMGB1 cytotoxicity was performed using 80 nM HMGB1 concentrations (72 h, n=3). (E) Interferon gamma concentration was determined by ELISA (see Experimental Procedures) and used as a positive control for the activation of the cultured and stimulated NK cells derived from blood donors. (F) Western blotting or quantitative PCR (G) was performed after treatment with HMGB1 (80 nM, 24 h). (H) Uncropped version of the silver gel showing bands after loading 0.5 µg and 0.1 µg protein respectively. *p<0.05, **p<0.002 (t-test). Error bars represent the SD.

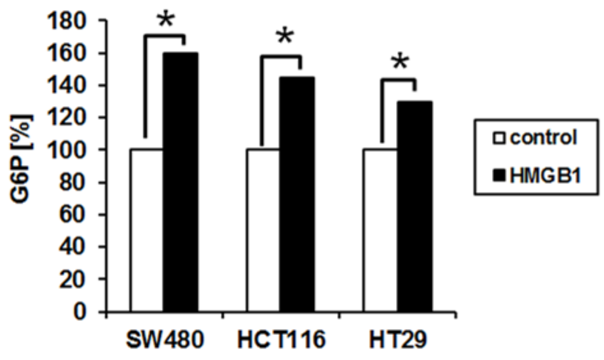

Supplementary Figure 2

HMGB1 causes accumulation of glucose intermediates up-stream of PKM2 by interacting with PKM2 near its allosteric center. Treatment with HMGB1 (80 nM, 24 h, n=3) resulted in accumulation of glucose-6-phosphate (G6P), the product of the hexokinase reaction. *p<0.05 (t-test). Error bars represent the SD.

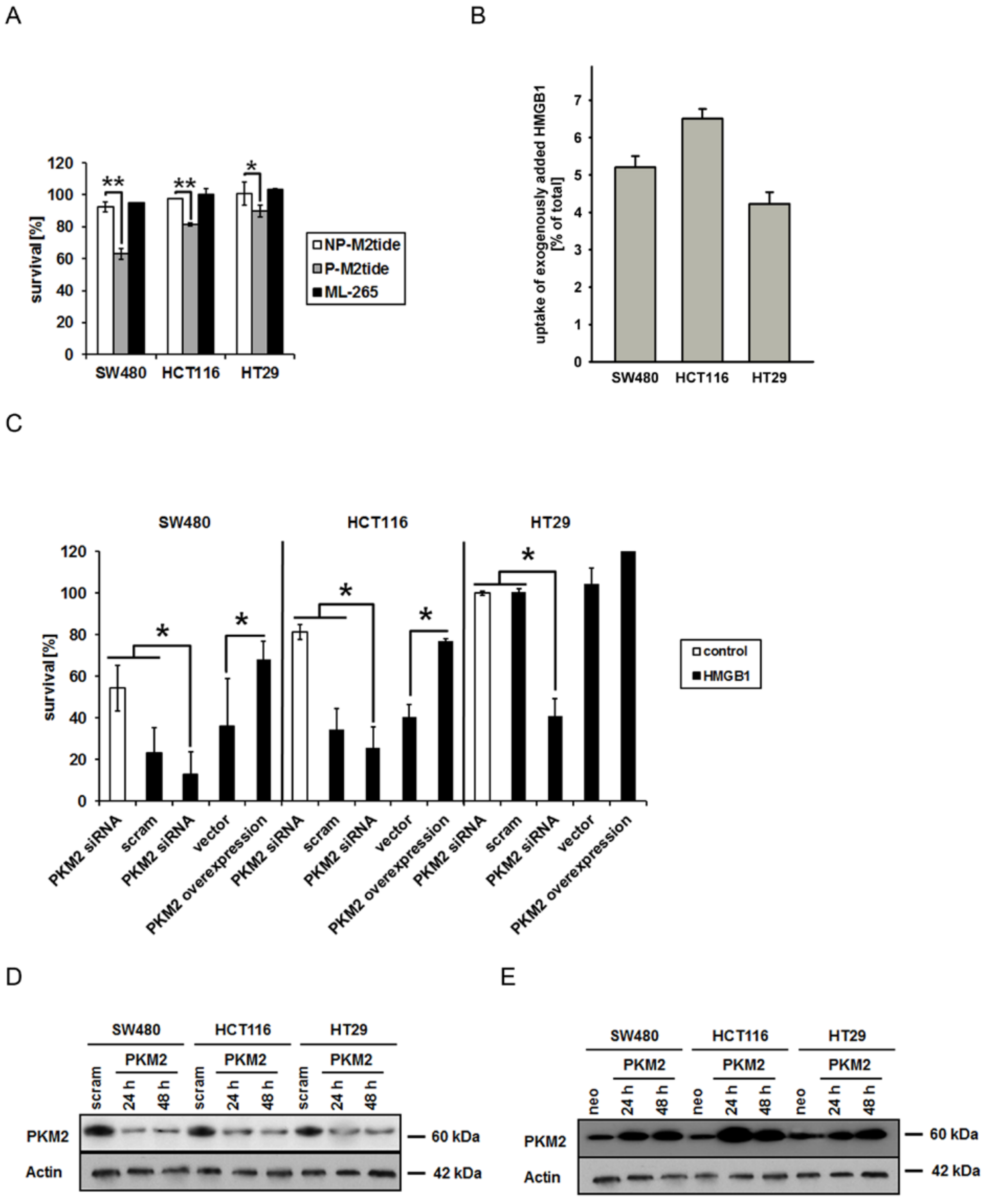

Supplementary Figure 3

Inhibition of tetrameric PKM2 phenocopies the cytotoxicity of HMGB1. (A) Cells were treated with 100 µM P-M2tide (phosphotyrosine peptide) for 24 h (n=3). The PKM2 activating small molecule ML-265 that binds to the dimer-dimer interface distant from the P-M2tide binding site did not induce

cytotoxicity. Non-phosphorylated P-M2tide (NP-M2tide) suited as a negative control. (B) Cytosolic fractions of the indicated cells treated with ^{125}I-labelled HMGB1 (80 nM, 24 h, n=3). (C) PKM2 was down-regulated or overexpressed transiently and then treated with HMGB1 (80 nM, 72 h, n=3). Successful knock-down or overexpression of PKM2 was confirmed by western blotting (D, E).

Error bars represent the SD. *p<0.05, **p<0.01 (t-test).

A

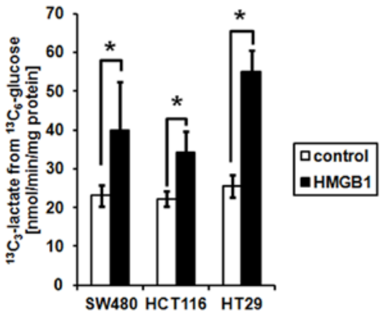

B

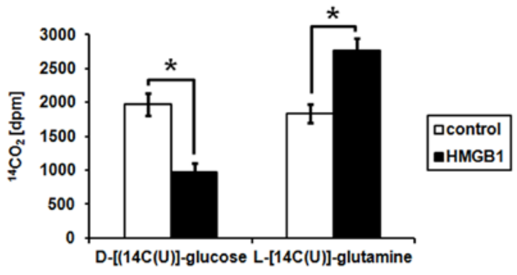

C

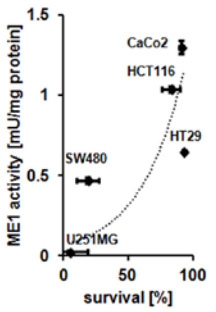

D

Feature	n (%)
Age at diagnosis	
Median	69
Range	33-94
Neoadjuvant therapy	
No	1170 (93)
Yes	90 (7)
Sex	
Male	718 (57)
Female	541 (43)
Grade of malignancy	
G1	20 (2)
G2	852 (68)
G3	351 (28)
G4	6 (<1)
GX	22 (2)
Tumor extent (TNM 2010)	
pT1	79 (7)
pT2	218 (19)
pT3	735 (63)
pT4	134 (11)
Lymph node metastasis	
pN0	636 (55)
pN1	287 (25)
pN2	231 (20)
Distant metastasis	
pM0	1085 (86)
pM1	175 (14)
UICC	
Stage 1	244 (19)
Stage 2	415 (33)
Stage 3	426 (34)
Stage 4	175 (14)
Localization	
Colon	806 (64)
Rectum	454 (36)
Family history	
No	1073 (86)
Yes	180 (14)

E

Feature	Hazard ratio (95% CI)	p
Age*	1.48 (1.31-1.67)	<0.0001
Chemotherapy	0.69 (0.51-0.93)	<0.02
Radiotherapy	1.04 (0.72-1.51)	0.8
ME1 expression positive vs negative†	1.24 (0.54-2.84)	0.6
ME1 expression strong positive vs negative‡	0.95 (0.47-1.92)	0.9
HMGB1 expression high vs low™	0.67 (0.52-0.87)	0.7
Sex§	1.10 (0.88-1.37)	0.4
Grade of malignancy¶	1.25 (0.99-1.57)	0.06
Tumor extent pT2 vs pT1	0.90 (0.44-1.86)	0.8
Tumor extent pT3 vs pT1	1.58 (0.82-3.04)	0.2
Tumor extent pT4 vs pT1	2.00 (0.99-4.06)	0.05
Lymph node metastasis pN1 vs pN0	1.97 (1.42-2.74)	<0.0001
Lymph node metastasis pN2 vs pN0	3.40 (2.44-4.73)	<0.0001
Distant metastasis€	4.49 (3.41-5.92)	<0.0001
Localization‖	1.16 (0.90-1.49)	0.3

Abbrevations:
ME1=malic enzyme 1; 95% CI=95% confidence intervall.
*10-years increment.
†Immunohistochemical score 3-6 vs <3.
‡Immunohistochemical score >6 vs <3.
™ Immunohistochemical score >6 vs 1-6.
§ Female vs male.
¶ G3/G4 vs G1/G2.
€ pM1 vs pM0.
‖ Rectum vs colon.

F

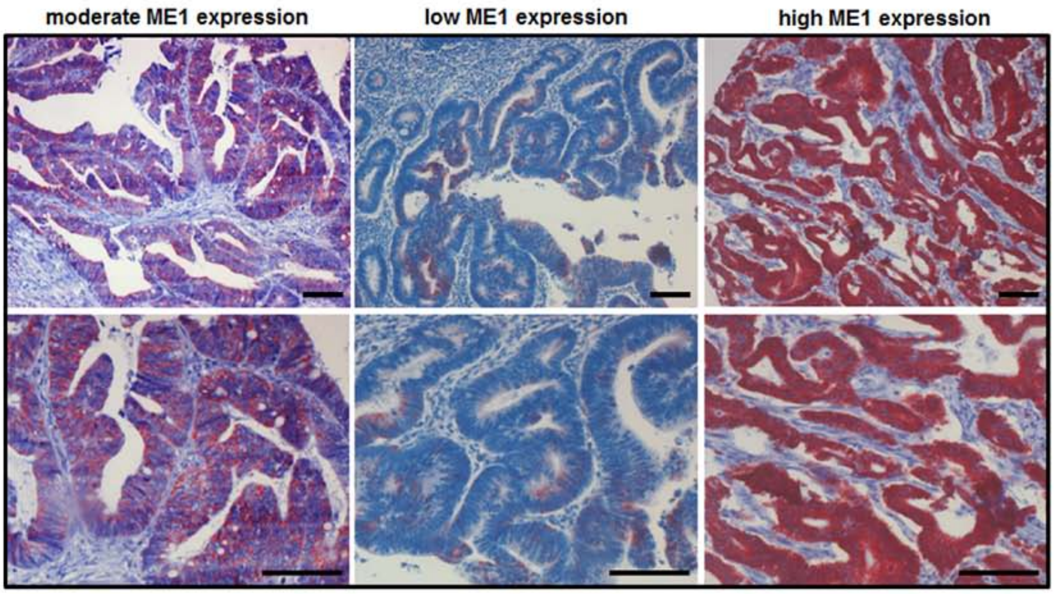
moderate ME1 expression
low ME1 expression
high ME1 expression

G

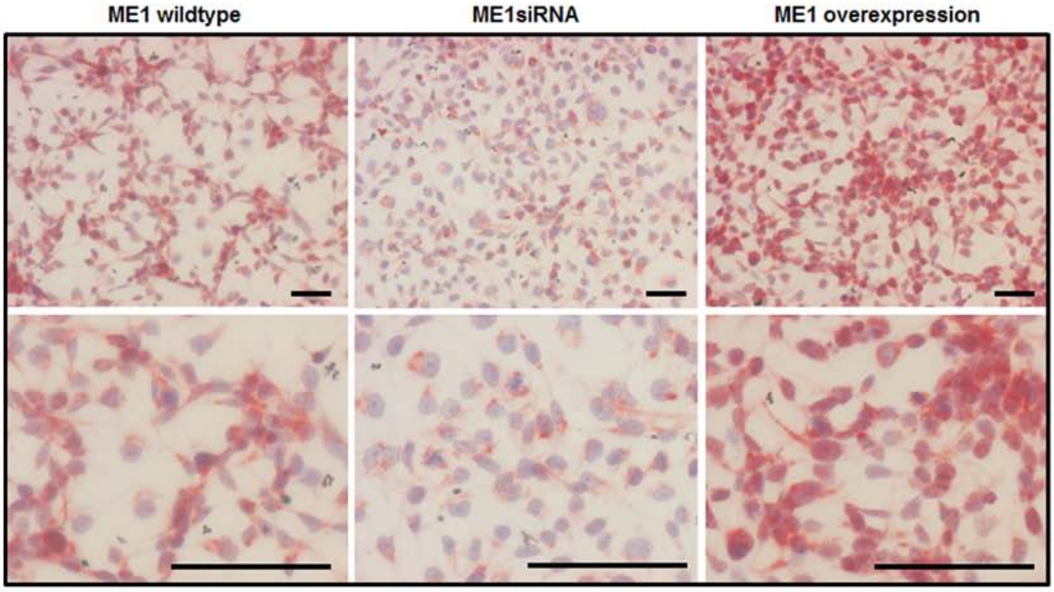
ME1 wildtype
ME1siRNA
ME1 overexpression

H

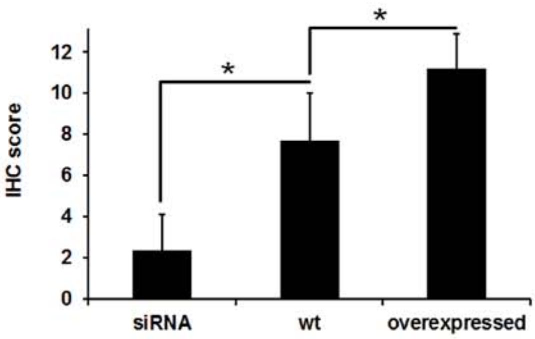
IHC score
12
10
8
6
4
2
0
*
*
siRNA
wt
overexpressed

I

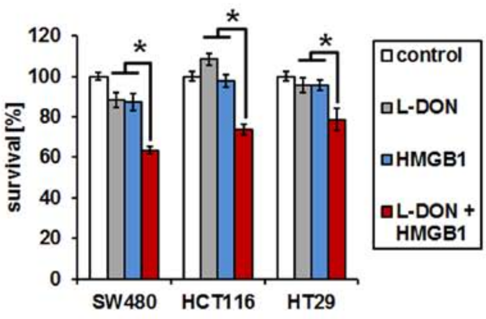
survival [%]
120
100
80
60
40
20
0
*
*
*
SW480
HCT116
HT29
control
L-DON
HMGB1
L-DON + HMGB1

J

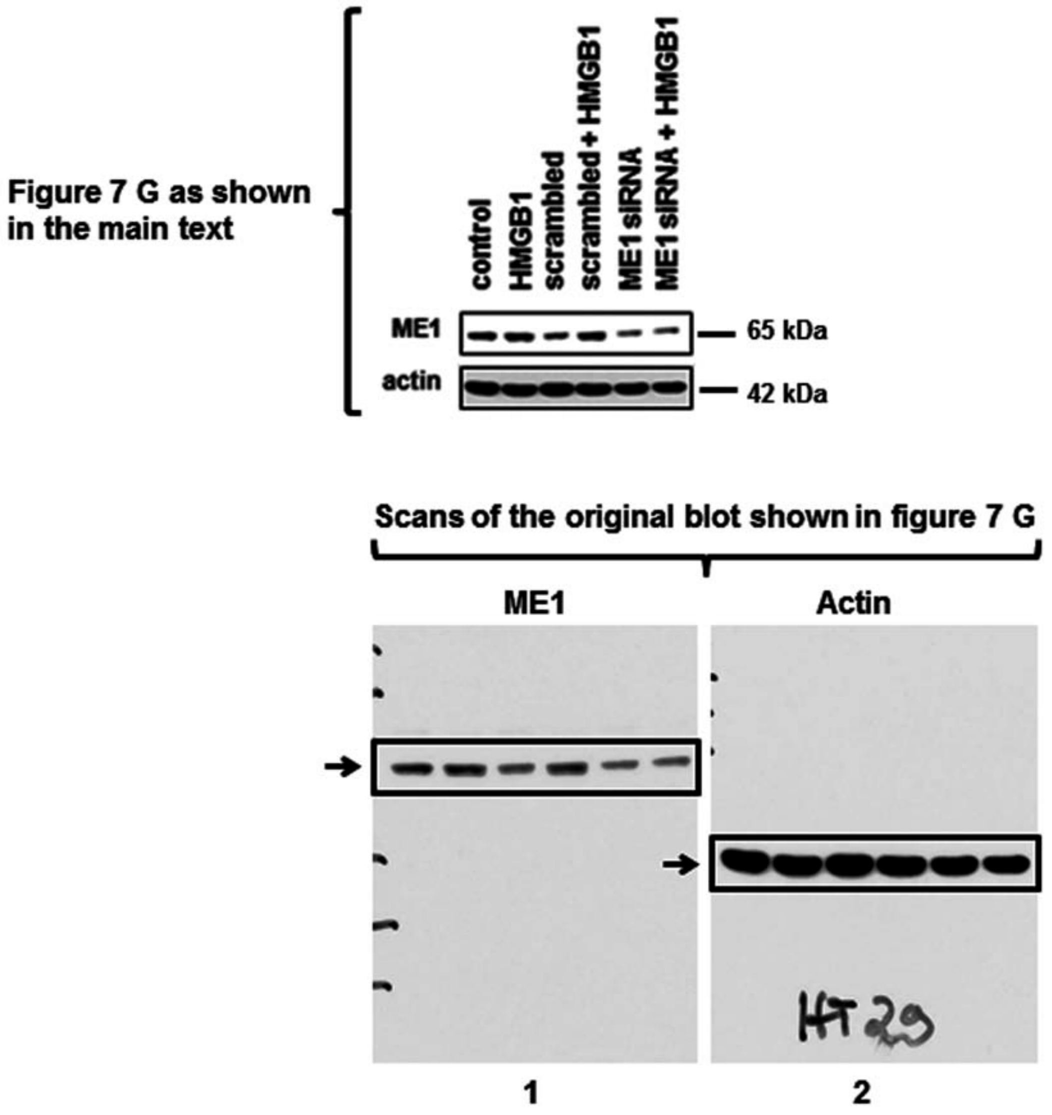

Supplementary Figure 4

Enhanced glucose fermentation and glutaminolysis define HMGB1 resistant cancer cells. (A) Lactate production from glucose was measured by tracking the ^{13}C-lactate efflux into the supernatant after treatment with HMGB1 (80 nM, 24 h, n=3, *p<0.04, t-test). (B) Production of labeled CO_2 from L-[^{14}C(U)]-glutamine or D-[(^{14}C(U)]-glucose in HT29 cells after treatment with HMGB1 (80 nM, 24 h, n=3, *p<0.002, t-test). Released $^{14}CO_2$ was trapped with a filter paper soaked with Hyamine. The filter papers were transferred to LSC vials mixed with 10 ml Ultima Gold LSC cocktail and counted for radioactivity. (C) ME1 activity was measured by monitoring NADP reduction in a buffer containing 250 mM saccharose, 50 mM KCl, 5 mM $MgCl_2$ and 20 mM Tris / HCl, (pH 7.4) using 2 mM malate and 0.5 mM NADP. Specific activities were measured before and after treatment with HMGB1 (80 nM, 72 h,

n=3). The basal specific activity of ME1 was slightly higher in untreated HCT116 (1.04±0.02 mU/mg) and HT29 (0.65±0.01 mU/mg) colorectal cancer cells compared to the HMGB1-sensitive colorectal cell line SW480 (0.47±0.02 mU/mg). Accordingly, the highly HMGB1-sensitive gliobastoma cell line U251MG [1] showed no detectable ME1 activity. Sequencing of the *me1* gene in these cell lines revealed no mutations (see Supplementary Data for the sequencing procedure, results not shown). Kinetic parameters of ME1 under physiological malic acid concentrations (0.02 - 0.2 mM) for HT29 cells displayed the lowest Km value (SW480: 0.074 mM, HCT116: 0.130 mM, HT29: 0.049 mM). (D) Summary of clinical and pathological features. History of a colorectal cancer in a first degree relative was defined as a positive family history for colorectal cancer. UICC (***U**nion **I**nternationale **C**ontre le **C**ancer,* International Union Against Cancer) stage grouping according to the WHO classification of tumors is pT1/pT2, N0, M0 for stage 1; pT3/pT4, N0, M0 for stage 2; any pT, pN1/pN2, pM0 for stage 3 and any pT, any pN, pM1 for stage 4. (E) Multivariate Cox regression analysis of ME1 and HMGB1 expression and clinical/pathological features for the prediction of overall survival in patients with colorectal cancer. (F) Representative ME1 images from the TMA showing cytosolic staining. Scale bars, 100 µm. (G, H) The specificity of the ME1 antibody was demonstrated in U251 cells; successful overexpression or down-regulation was confirmed by western blotting. Scale bars, 100 µm. (I) Crystal violet survival assay in glucose supplemented medium after treatment with HMGB1 (80 nM, SW480 and HCT116; 160 nM, HT29; 24 h). L-DON (1 µM) was added as indicated (n=3, *p<0.05, t-test). (J) shows the original blot probed for ME1 (1) or actin (2); HT29=HT29 cell line.
Error bars represent the SD.

SUPPLEMENTARY REFERENCES

1 Gdynia, G. *et al.* Danger signaling protein HMGB1 induces a distinct form of cell death accompanied by formation of giant mitochondria. *Cancer research* **70**, 8558-8568, doi:10.1158/0008-5472.CAN-10-0204 (2010).
